# Chr:17q21.31 locus risk haplotype H1 susceptibility to ferroptosis is mediated by endolysosomal pathway

**DOI:** 10.1038/s41419-025-08147-1

**Published:** 2025-11-13

**Authors:** Eldem Sadikoglou, Daniel Domingo-Fernández, Natalia Savytska, Noemia Fernandes, Patrizia Rizzu, Anastasia Illarionova, Tabea Strauß, Sigrid C. Schwarz, Alpha Kodamullil, Günter U. Höglinger, Ashutosh Dhingra, Thomas Gasser, Peter Heutink

**Affiliations:** 1 German Center for Neurodegenerative Diseases (DZNE), Tübingen, Germany; 2 Fraunhofer Institute for Algorithms and Scientific Computing, Sankt Augustin, Germany; 3 German Center for Neurodegenerative Diseases (DZNE), Munich, Germany; 4 Department of Neurology, Technical University Munich, Munich, Germany; 5 curiositas-ad-sanum GmbH, Haagi, OB Germany; 6 Department of Neurology, LMU University Hospital, Ludwig-Maximilians-Universität (LMU) München, Munich, Germany and Munich Cluster for Systems Neurology (SyNergy), Munich, Germany; 7 Hertie Institute for Clinical Brain Research, Tübingen, Germany and Department of Neurology, University Hospital, Tübingen, Germany

**Keywords:** Cell death in the nervous system, Neurodegenerative diseases, Cell death, Genetics research, Cellular imaging

## Abstract

Human chr:17q21.31 locus is a complex genomic region of high linkage disequilibrium with two main haplotypes, named H1 and H2. The H1 haplotype is genetically associated with a wide spectrum of neurodegenerative diseases (NDs), including tauopathies and synucleinopathies, with the underlying mechanism remaining unknown. We investigated the interplay of environmental and genetic risk factors on neurons derived from iPSCs of both haplotypes under Mild Chronic Oxidative Stress (MCOS) conditions. The observed increased susceptibility of H1 neurons to MCOS leading to an earlier neuronal death, was mediated by ferroptosis. Characterization of the phenotype revealed spatiotemporal propagation and spreading of axonal deterioration and neuronal death in accordance with NDs pathology. Transcriptional profiling pointed to ferroptosis hallmarks and endo-lysosomal vesicles as implicated pathways, while FDA-approved drugs prevented the induced death in H1 neurons. Finally, ROS and lysosomal dynamics during the neuronal maturation shed further light to the differential response of haplotypes to MCOS, which could explain the risk association of the H1 haplotype with NDs.

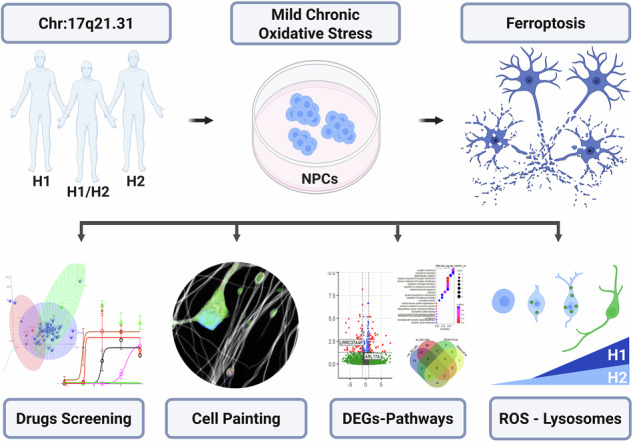

## Introduction

Genome Wide Association Studies (GWAS) have repeatedly demonstrated a strong correlation between the *MAPT* (chr:17q21.31) locus and neurodegenerative diseases (NDs), although the causal gene(s) and genomic variants have not yet been identified. The chr:17q21.31 locus occurs as two major haplotypes, H1 and H2 harboring a 970-kb inversion, thousands of single nucleotide polymorphisms (SNPs), duplications or microdeletions [1–3]. Locus genes other than *MAPT* are the Corticotropin releasing hormone receptor 1 (*CRHR1*), Saitohin (*STH*), the N-Ethylmaleimide Sensitive Factor-Vesicle Fusing ATPase (*NSF*), and others [4–6]. H1 is the major ancestral haplotype, while the H2 allele is present only in 20% of Caucasian origin Europeans, is rare in Africans, and almost absent in East Asians [7]

The H1 haplotype consists an increased risk for a diverse group of NDs, including tauopathies such as Progressive Supranuclear Palsy (PSP), Corticobasal Degeneration, APOE ɛ4-negative Alzheimer’s disease (AD), Frontotemporal Dementia [8–14], synucleinopathies like Parkinson’s disease (PD) [15, 16] and Dementia with Lewy bodies [17], the Multiple System Atrophy and even Essential Tremor (ET) [18] and Amyotrophic Lateral Sclerosis (ALS) [19]. Whereas the H2 haplotype has been associated with the chr:17q21.31 microdeletion syndrome [20]. Although initially studies focused on the differential expression of MAPT [5, 21–23], later studies proved that other genes within the locus were differentially expressed as well [1, 2, 6, 24]. Despite the strong genetic association of the locus with NDs, up until today there is no conclusive functional experimental evidence linking the H1 haplotype with ND related molecular signatures.

Consequences of oxidative stress (OS) in the pathophysiology of NDs [25] have been extensively studied by direct or indirect induction of reactive oxygen species (ROS) [26–30]. Excessive ROS cause axonal fragmentation, cytoskeletal-microtubule disassembly, and neuronal death [27, 29, 31–34]. However, the majority of these studies were conducted with acute exposures to high concentrations of chemicals of already differentiated primary neurons from rats or mice carrying the H1 haplotype [27, 31, 32, 34].

In the present study, we examined the impact of Mild Chronic Oxidative Stress (MCOS), as a more relevant model to the pathophysiology of NDs, on the genetic background of H1-H2 haplotypes. Neural progenitor cells (NPCs) derived from nine iPSC lines of healthy donors carrying the H1, the H2 homozygous, and the H1/H2 heterozygous haplotypes were used [35]. The induced MCOS, after removing antioxidants (AO) from NPCs and neurons for ~1.5 months, caused extended signs of axonal degeneration prior to neuronal death, similar to those described before [27, 28, 34, 36]. Furthermore, we report here for the first time a human haplotype-specific difference in susceptibility to MCOS with the H1 risk allele being more sensitive than the H2. Neuroprotective agents, after the utilization of FDA-approved molecules screening, in combination with time-lapse imaging, revealed insights regarding the mechanism of the observed cytotoxicity. MCOS induced propagation of neuronal death was due to ferroptosis, the recently described iron mediated lipid peroxidation dependent cell death mechanism [37, 38], that is strongly associated with NDs [39]. Morphological and transcriptional profiling of cells, harboring both haplotypes, under MCOS uncovered the differential implication of the endo-lysosomal system with the phenotype. Our results were supported by evidence from longitudinal study of ROS and lysosomal dynamics during the neuronal maturation of H1 and H2 haplotypes.

## Materials and Methods

### Cell culture—Mild chronic oxidative stress model

All iPSCs (three per haplotype) were obtained from healthy individuals from HipSci collection (Human Induced Pluripotent Stem Cell Initiative) and were characterized in our previous study [35]. All cell lines were regularly checked for mycoplasma contamination (Venor GeM Classic, TH-GEYER) and genotyped for the haplotype with PCR detection of 238 bp deletion on intron 9 of MAPT gene [40].

Small molecule Neuronal Progenitor Cells (NPCs) derivation from iPSCs and expansion was done as before [41]. NPCs were transduced with *Neurogenin* 2 (*NGN2*) containing Lentivirus [42], expanded after selection with Blasticidine (ant-bl-05, InvivoGen) and cryopreserved in liquid Nitrogen.

MCOS conditions were triggered by culturing NPCs for indicated weeks and seeded for neuronal differentiation with media lacking known antioxidants like vitamin E, glutathione, superoxide dismutase, and catalase (B27 suppl. minus Antioxidant, 10889038, Gibco). For rescue experiments, the minusAO media was replaced with media that contained either plusAO or FDA-approved molecules on day6 of neuronal differentiation.

### Neuronal differentiation

Equal numbers of NPCs treated with and without AO, were seeded and differentiated into neurons on Poly-L-Ornithine-laminin coated 96 black well plates (PerkinElmer) with 2.5 µg/mL doxycycline and 10 µM DAPT according to others [35]. Complete media changes on day3 and day6 of neuronal maturation with N2B27 media supplemented with neurotrophic factors like brain-derived neurotrophic factor (BDNF), glial cell-derived neurotrophic factor (GDNF), neurotrophic factor 3 (NT-3), 10 ng/mL each, and 0.05 µg/mL Laminin was performed with minimum flow rates and VIAFLO electronic pipettes (INTEGRA).

Throughout the study, the labelling was done on live neurons at different maturation stages with bio-probes and concentrations indicated in Table 1 according to manufacturer’s instructions. For the Cytotoxicity assay, Spectramax M2 Multimode Microplate Reader (Molecular Devices) and Cell Voyager Yokogawa microscope (CV7 000, Tokyo, Japan) were used.

**Table 1:** Bio-Fluorescence probes for live neuronal labelling.

Assay	Fluorescence probe	Target	(nm)	O.C. mM	F.C. μM	Num. Vendor
Screening	Calcein-AM Red	Membranes	577/590	1	0.1	C34851, ThermoFischer
Time-lapse	LIVE/DEAD kit	Membr./Nuc.	494/517	1	0.1	L3224 ThermoFischer
Cell painting assay	Liperfluo	Oxidized Lipids	488/550	1	1	L248, Dojindo
	MitoTracker-Red	Mitochondria	579/599	1	0,1	M7512, ThermoFischer
	Calcein-AM Green	Membranes	488/550	1	0.5	C34852, ThermoFischer
	Hoescht 33342	Nuclei	350/461	16.2	2	H3570, ThermoFischer
	LysoTracker-Red DND-99	Lysosomes	577/590	1	0.1	L7528, ThermoFischer
	Tubulin Tracker	Microtubules	652/669	1	1	T34077, ThermoFischer
Longitudinal assay	pHLys-Red	Lysosomes	560/590	1/1000		L265, Dojindo
	ROS	Total ROS	505/525	10	2	R253, Dojindo

### Primary and secondary screens with FDA-approved chemicals library

The FDA-approved Drugs Library (L1300, Selleckchem) obtained by Hoelzel Diagnostika Handels was diluted at 1 mM in DMSO and stored at −80 °C in 96 deep well plates. Before use, compound plates were equilibrated to room temperature. Screening plates were handled in a semi-automated system with VIAFLO 96/384 handheld electronic pipette (INTEGRA Biosciences) and Multi-Mode Dispenser MultiFlo FX (BioTek Instruments). Screening assays were performed with −5wAO depleted H1 cell line (HPSI0913i-diku_1) differentiated to neurons with standard day3 media change without AO. On day6 media change, minusAO was replaced with plusAO media for positive control, whereas minusAO media was used as negative control. Both controls include 0.5% DMSO (see supplementary file). Primary screening was performed in two independent batches and in total 1430 compounds spread across 30 plates (Fig. S2D) were screened in *n* = 4 replicas. 5 μM was the concentration of drugs used for primary screen (see supplementary file Fig. S2B) and the cell painting assay, while five-fold serial dilutions (from 5 μM to 8 nM) were used for the dose response curve assay. At the end of treatments live neuronal staining was performed with Calcein-AM Red addition to the media and incubation for 30 min at 37 °C. Each batch of primary screen included 60×96-well plates with 3600 wells and 15 fields per well were imaged on confocal microscope Cell Voyager (CV7000, Yokogawa). Live neuronal counts determined after image analysis on CellPath Finder (version 3.03.02.02, Yokogawa) were analyzed with open-access web-tool for High Throughput Screening applications, HitSeekR [43] using plate-wise normalization. The *n* = 4 replicates correlation with corresponding R^2^ from the analysis are shown in supplementary file (Fig. S2E)

Dose response curve experiment and the Cell Painting assay were done by cherry picking manually the primary hits from the library and reformatting into deep well plates with minusAO and plusAO controls included as before. Five-fold serial dilutions (from 5 μM to 8 nM) were prepared into new deep well plates and transferred to neurons during day6 media change with VIAFLO 96/384. Cell staining and imaging was performed as before.

### Pathway enrichment with primary hits-protein targets

Two complementary approaches were used to identify chemical-protein interactions of primary hits. In the first approach, we relied on well-established interactions from DrugBank that were further enriched via manual curation. On the other hand, the second approach employed a text mining engine (SCAIView; https://academia.scaiview.com) to systematically extract chemical-protein interactions described in scientific literature. Next, by using the interactions retrieved from both approaches, we mapped proteins that interact with each of the primary hits to pathways in three major databases (i.e., KEGG, Reactome, and WikiPathways). The equivalent pathways were grouped together using their pathway hierarchy as well as the mappings from ComPath [44]. Additionally, we included a gene set curated from FerrDb (V1, 2020) representing ferroptosis, as it was either not integrated or had only few annotated genes in the major databases.

### Image acquisition and analysis

All imaging assays were performed on live neurons with indicated fluorescence bioprobes (Table 1). Image acquisition except the brightfield images, was done on Cell Voyager spinning disk confocal CSU imaging system CV7000 (Yokogawa). Stage incubator with 5% CO_2_ and 37 °C temperature was used for all imaging experiments.

Primary screen assay was imaged with 10× air objective in single z-stack and 15 fields per well, producing in total 900 images per plate in seven minutes and 54.000 images per run. Secondary screening of the dose response curve experiment 14 fields of single z-stack were used per well for all concentrations except the 5 μM which was imaged in z-stacks to be used in TAL analysis. Time-Lapse images of 5h and 24h were imaged every 20 and 60 min, respectively.

Throughout the study, image analysis was done mainly with CellPathfinder (v3.03.02.02), whereas blebs counting in Cell Painting assay was performed with deep learning functions (v3.06.01). Neuronal counts of dose response curve experiment were done with the CellProfiler (v2.2.0), due to extensive clumping. ImageJ (2.3.0/1.53f51, Java 1.8.0_172 (64-bit)) was used occasionally to construct bright field image composites.

### Cell painting assay

After 48h and 72h of incubation with secondary hits, cells were stained with the fluorescence probes indicated in Table 1 on day8 and day9 of differentiation. The staining solutions were prepared as 4× concentrations in minusAO media, added to each well (50 µL), and incubated at 37 °C for 30 min. After incubation and before imaging half of the media was replaced. Each time point was carried out in *n* = 4 of replicate plates. The combination of bioprobes per day were determined in a preliminary experiment according to MCOS induced ferroptosis phenotype. We observed that the very first cellular compartments which were affected and destroyed were the mitochondria and the membrane lipids. Therefore, we set up the imaging of the above alongside the nucleus and the microtubules, on day8 of differentiation, the time point where we observe the earliest signs of stressed neurons. For day9 neurons, where we observed more advanced signs of axonal degeneration with extensive blebbing and dying neurons in minusAO treated controls, we used fluorescence probes targeting cell membrane integrity, lysosomes, nucleus, and microtubules.

Imaging conditions of degenerating neurons during the dying process were set to 2h per plate on Cell Voyager CV7000. We imaged 14 fields per well with 60× water immersion objectives and several z-stacks. Automatic dark and shading correction of images were done with CV Image Correction Tool R2.02.06. Cell Pathfinder (CPF) software.

Blur or damaged images were removed from analysis by using the Thumbnail function of CPF and simultaneously examination in a plate-wise manner of all fields. Machine learning function was used to identify nuclei (Hoechst channel) and by filter applications, they were separated into Live and Dead Nucleus (NucL. and NucD.) clusters. The live nucleus seeds were used as a reference region to identify the cell boundaries for the lipids and the oxidized lipids staining on day8 and the cell membranes on day9. Mitochondria of neuronal soma were excluded from analysis since they are difficult to segment and need special imaging techniques. Total Lysosomal numbers on day9 neurons were identified, while a deep red channel was used to detect the axons-microtubules for both days of imaging. The axonal length and the area covered by axon-related features were used in the analysis, as the junctions and branching counts cannot be determined accurately in cultures with advanced neural networks. Finally, day9 images were used for the identification and the counting of blebs on axons with deep machine-learning function and deep image gating with CellPathfinder (v3.06.01). All the above determined objects were used to measure and extract morphological features related to size, shape, texture, and intensity per field and per object. Additional mathematical expressions like the percentage of live nucleus counts versus the total number of nucleus identified were extracted and used for the analysis and the quality control in comparison between and among the plates. Pearson correlation was determined with positive controls among the plates to exclude possible batch effects as part of quality control [45]. In general, we extracted and used in dimensionality reduction analysis (PCA) more than 200 features per day of differentiation, after removing all the total values which were not averaged either per field or per cell number.

### RNA-seq and pathway enrichment analysis

RNeasy mini kit (Qiagen 74106) was used for RNA extraction from six-well format of day8 neurons treated and differentiated with plusAO and −5wAO as before. Libraries were prepared with PCR-cDNA sequencing—barcoding kit (Nanopore, SQK-PCB109) and run on PromethION24 (Nanoporetech). All RNA extraction and sequencing reactions run in parallel to eliminate batch effects. Raw reads processing were performed with nf-core/nanoseq v2.0.1 [46] using options “–protocol cDNA –flowcell FLO-PRO002 –kit SQK-PCB109 –barcode_kit SQK-PCB109 -profile singularity”. Mapping to reference genome GRCh38 was done with minimap2 v2.17 [47] while a combination of gencode v29 [48] and lncipedia v5.2 [49] annotations were used for gene annotation and quantification with Bambu v1.0.2 [50].

DEGs were determined using the R package DESeq2 v1.37.6 after filtering out Y chromosome and entries with minimum 2 samples sum of normalized counts less than 10. As background, all expressed genes were used. Significantly up and downregulated genes were determined with *p* < 0.05 and absolute log2 Fold Change >1. For volcano plots the EnhancedVolcano package, v1.16.0 [51], and for Venn diagram, the web tool (http://bioinformatics.psb.ugent.be/webtools/Venn/) was used.

Functional enrichment analysis was performed with multiple databases like the Gene Ontology database [The Gene Ontology Consortium, 2023] and a custom list with KEGG, Reactome, WikiPathways, and ferroptosis database, FerrDb (as of 25/09/2023). The R package ClusterProfiler v4.6.2 and the Comparecluster function was used in overrepresentation (ORA) and gene set enrichment analysis (GSEA), where *P*-values were set to <0.05 with no adjustment methods. Enrichment maps were plotted with ggplot2 v3.4.4.

STRING, v12.0 (https://string-db.org) was used for the protein-protein interaction network of the *ARL17A* (ADP Ribosylation Factor Like GTPase 17A), including all “Homo sapiens” related sources with size cut off 10 and confidence score > 0.4. The line color indicates the type of interaction evidence and the thickness indicates the strength of data support.

### Longitudinal assay

The MCOS treatment for the study of ROS and Lysosomal dynamics during neuronal maturation for both haplotypes was done for the indicated weeks as before. For ROS staining, an improved version [52] of dichlorofluorescin–diacetate, DCFD-DA (Dojindo, Japan) was used. The Lysosomal staining was performed with pHLys (Red, Dojindo Japan) that showed higher lysosomal specificity and sensitivity to pH changes than the widely used LysoTracker-Red DND-99.

Staining and imaging conditions were kept constant in time and weeks of treatment. Image analysis was processed in batch mode with CellPathfinder. Identified objects and features of three independent biological replicates per haplotype, three technical and five experimental repeats, each lasting approximately ~7 weeks, were analyzed with three way-ANOVA of repeated measures (3 way RM-ANOVA).

### Research tools and software

Statistical analysis was done with GraphPad Prism v9.1.0 for Windows (GraphPad Software, La Jolla, California, USA, www.graphpad.com). All figure composites prepared with Biorender (Created with BioRender.com) and the related permissions to publish figures/graphical abstract in journal were obtained. Primary screening analysis was performed with the open-access software for High Throughput screening application HitSeekR [43]. Chemical structure similarity was performed with the Java-based open source tool for the visual analysis and interactive exploration of chemical space, Scaffold Hunter v2.6.3 [53]. RStudio Team (2020) v4.2.1 and related packages like FactoMineR [54], Factoextra [55], ggplot2 [56], and rgl [57] were used for analysis and plots.

## Results

### Differential susceptibility of chr:17q21.31 locus haplotypes to MCOS

NPCs of H1, H2 and the heterozygous H1/H2 cell lines, treated with and without AO for several weeks, were differentiated into neurons and subjected to cytotoxicity assay. Although the NPCs were unaffected (Fig. S1B, C), a clear difference in viability between the minusAO treated neurons was observed (Fig. S1D–G). Moreover, an additive effect by prolonged weeks of AO depletion and increasing days of neuronal maturation was detected (see supplementary file and Fig. S1A).

**Fig. 1:**
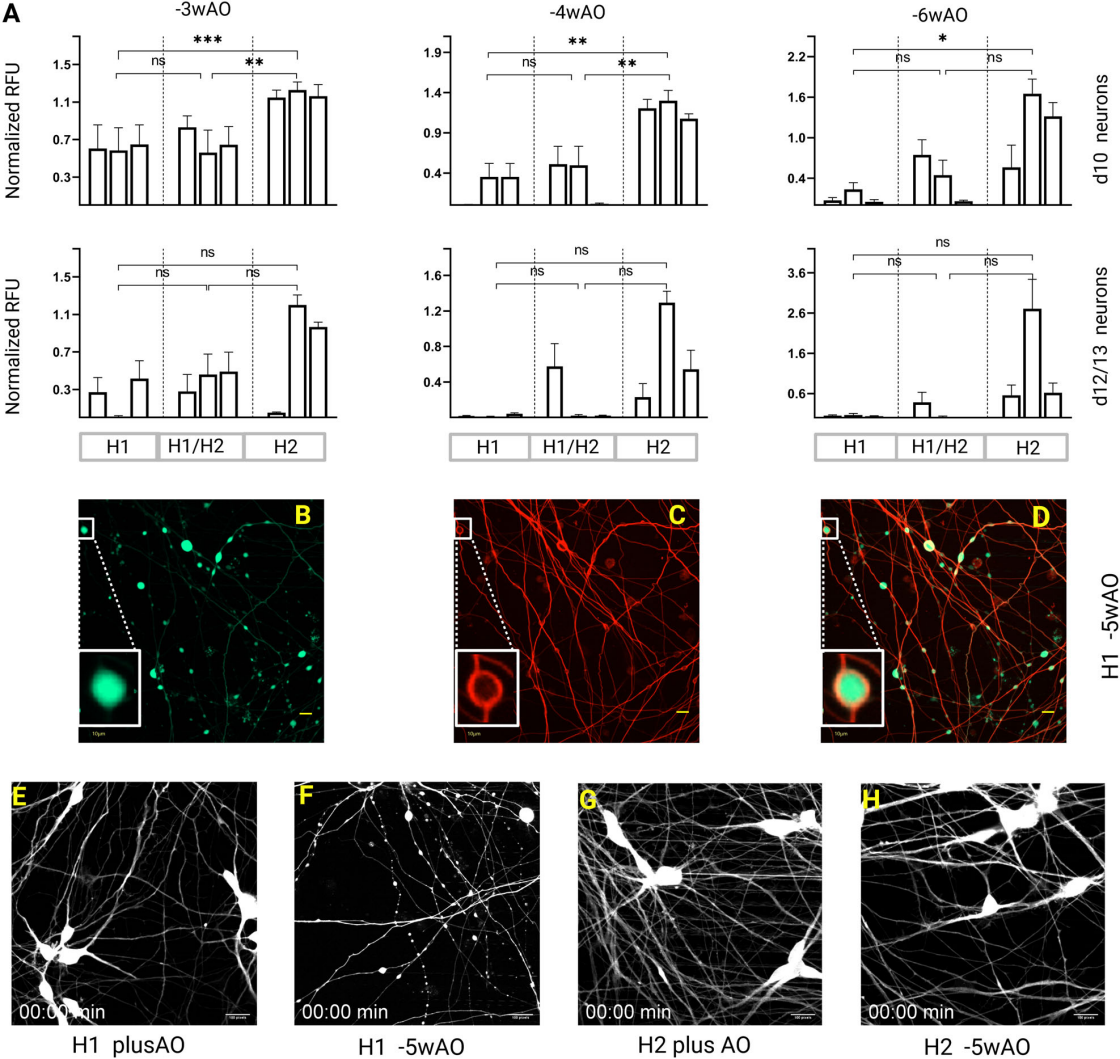
Chr:17q21.31 locus haplotypes susceptibility to mild chronic oxidative stress. **A** Relative fluorescence units of Calcein-AM (Red) viability assay with microplate reader. All nine cell lines treated in parallel for indicated weeks without AO were normalized to plusAO treated cells and analyzed with nested one-way ANOVA and Tukey’s post hoc test. Data represent mean and ± SEM of three biological replicates per haplotype, in three technical replicates and two independent experimental repeats. *P*-values are 0.1234 (ns), 0.0332(*), 0.0021(**), 0.0002(***). **B**–**D** Representative fluorescence images of H1 −5wAO treated day8 neurons with Calcein-AM green (**B**) and Tubulin Tracker™ far-red (**C**). Blebs and disarranged regions of microtubule bundles overlap in (**D**). Scale bar 10 μm. (**E**–**H**). Representative frames from time lapse images for 5h (3fph) of day8 neurons with Calcein-AM of H1 (**E** and vS01, **F** and vS02) and H2 (**G** and vS03, **H** and vS04) haplotypes treated with (**E**–**G**) and without AO for 5weeks (**F**–**H**). MCOS triggered blebs formation on degenarating axons and neuronal death in (**F**). Scale bar 100 pixels on ImageJ. For complete time-lapse videos refer to supplementary videos vS01 to vS04.

To assess whether this difference was significantly correlated to the haplotypes, the average fluorescence units of minusAO treated neurons were analyzed with nested one way-ANOVA (Fig. 1A) after normalization to plusAO. At −3wAO depletion, day10 neurons of the H1 haplotype were significantly more sensitive to MCOS than the H2 (*p* < 0.0002). The difference became less significant with increasing time of treatment (*p* < 0.002 for −4wAO and *p* < 0.033 for −6wAO), since some neurotoxicity was observed in H2 lines as well. The heterozygous H1/H2 cells were significantly more sensitive than the H2 at −3wAO and −4wAO treatment (*p* < 0.002). In developmentally more advanced neurons (days 12/13), despite the obvious differences between haplotypes, the significance was lost due to affection of H2 lines as well.

In summary, the observed neurotoxicity was correlated to AO depletion in a time and dose-dependent manner. Detailed time course of rescue with AO was done for the screening assay setup (see supplementary file and Fig. S2B)

### MCOS induced axonal degeneration and ferroptosis

We observed extensive signs of axonal degeneration prior to neuronal death after AO depletion, similar to findings in human tauopathies [58]. Blebs, also referred to as swellings, spheroids, or varicosities [28, 29, 59] appeared in −5wAO treated H1 neurons (Fig. S1E). Co-staining membranes with Calcein-AM (Fig. 1B) and microtubules with tubulin tracker (Fig. 1C) showed extensive overlap of blebs with disorganized microtubule bundles (Fig. 1D). In time-lapse imaging (supplementary videos vS01 to vS04), the progression of axonal deterioration over time was evident. Blebs in H1 −5wAO neurons (Fig. 1F, vS02) started as small elongated spots on axons, increased in size and numbers while axons thinned gradually before the neuronal death. Evidence of the above cascade of events, other than some transient swellings due to big cargo transport movements, were absent in H2 −5wAO (Fig. 1H, vS04) and the plusAO H1 and H2 neurons (Fig. 1E, vS01 and G, vS03) treated in parallel.

An intriguing observation was the spatiotemporal progression of neuronal death, which differed from other regulated cell death models [60]. The axonal degeneration and the subsequent death were spreading and propagating in a wave-like manner, which was reported earlier as a characteristic feature of ferroptosis [61, 62]. By recording live/dead neurons of both haplotypes at −5wAO on days 9, 12, and 21 of neuronal maturation for 24h, in tiled fields we showed the initiation and spreading of neuronal death in the cellular population (see supplementary Fig. S1H–M and videos vS09 to vS14) that was earlier for H1 than the H2 cell lines, in agreement with our neurotoxicity assay (Fig. 1A).

### Small molecules library screening and structure similarity analysis of hits

After elucidating the differential sensitivity to AO depletion between the H1 and H2 haplotypes, we established a screening assay (see supplementary file and Fig. S2) to identify small molecules that could reverse the detrimental effect of MCOS on H1 neurons.

**Fig. 2:**
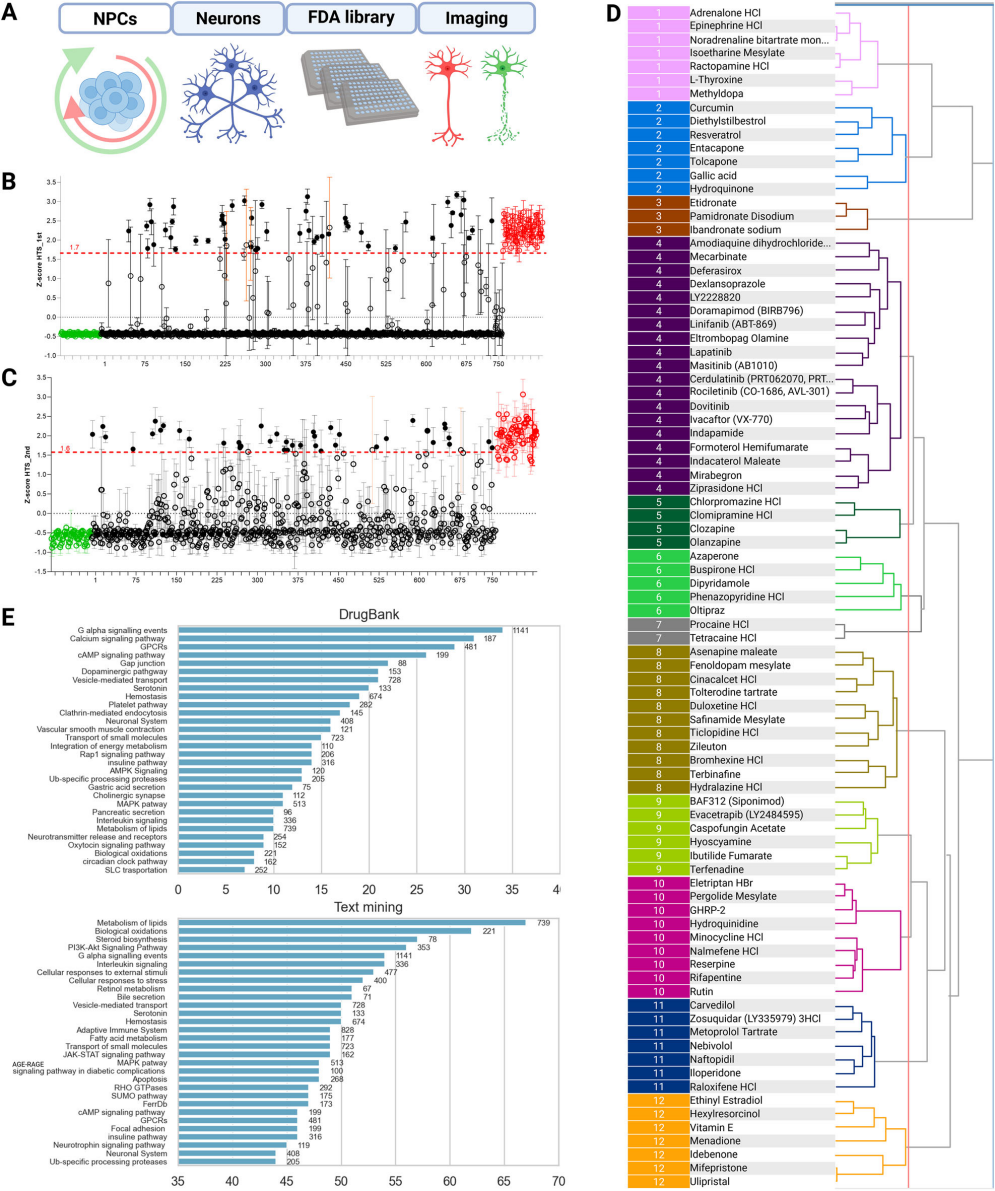
FDA-chemicals library screening and primary hits inspection. **A** Screening assay scheme with −5wAO depleted NPCs derived H1 neurons, rescued with FDA-approved drugs on day6 and imaged with Calcein-AM on day12. **B**, **C** z-scores of chemicals into two batches (HitSeekR). Green and red circles represent negative and positive controls, respectively. Among chemicals (black circles), hits are black dots above the cut off (red dotted lines). Hits with SD > 0.7 from *n* = 4 replicates (orange error bars) were excluded from the list. **D** Exact SAHN clustering dendrogram of primary hits based on their DayLight fingerprints and Ward’s linkage methods (Scaffold Hunter) returned 12 diverse clusters of structurally similar drugs. **E** Enriched pathways among primary hits protein targets obtained from DrugBank or the literature with text mining (SCAIView, Jun 2022). Both sets were mapped to KEGG, Reactome, WikiPathways and grouped together using their pathway hierarchy. On *Y*-axes 30 of the most enriched pathways, on *X*-axes the number of hits targeting each pathway.

The screening protocol (Fig. 2A) included −5wAO treated neurons rescued on day6 of neuronal maturation with AO or drugs. A library of FDA-approved chemicals (Selleckchem) was utilized into two batches and analyzed with HitSeekR [43]. An overall hit rate of 6.7% was obtained with thresholds at *z*-scores >1.7 × SD and >1.6 × SD of positive control (Fig. 2B, C). Inclusion of numerous ferroptosis inhibitors like, Deferasirox, Dexlansoprazol, Zileuton, Carvedilol, and others [63–65], among primary hits confirmed our earlier observations regarding the cell death mode. In contrast, the apoptosis (pan-caspase) inhibitor Emricasan, [66], a number of autophagy inhibitors (Metformin, Nimodipine, Azithromycine, Chloroquine, Hydroxy-chloroquine) [67, 68] and two forms of vitamin C failed to prevent the MCOS induced neurotoxicity.

Chemical structure similarity analysis has proven to be a useful tool in hit prioritization after high-throughput screening assays [69]. Clustering of primary hits with the Sequential Agglomerative Hierarchical Non-overlapping (SAHN) method [53] returned 12 distinct groups (Fig. 2D), in agreement with the structural diversity of AO molecules. Detailed examination of clusters (see supplementary file and Tables S1 and S2) showed various known natural AO compounds grouped under different clusters. The first two clusters consisted mostly of polyphenolic compounds like phenylalanine derivatives (Cluster 1) and stilbenes with flavonoids (Cluster 2). Cluster 4 was the most diverse group with antineoplastic, anti-inflammatory drugs, kinase inhibitors, quinolones, and indoles with known AO activities (see supplementary file, Tables S1 and S2)

### Enriched pathways among primary hits

Structure similarity analysis and molecular targets of primary hits (Tables S1 and S2) demonstrated a wide range of chemical structures and molecular activities. In order to gain further insights on the pathways targeted by the primary hits, we performed enrichment analysis by extracting chemical-protein interactions from DrugBank [70] and from the literature with a text mining engine (SCAIView https://academia.scaiview.com) (Fig. 2E). The text mining approach, which includes more recent research data compared to DrugBank, yielded a larger number of protein targets per primary hit. Interestingly, the targeted pathways and their hierarchy based on the number of hits varied significantly between the two approaches.

The most targeted pathways in DrugBank-interactions were the G alpha and the Calcium signaling pathways, with 30 hits targeting each one of them. In the text mining approach, the most targeted pathways were the lipid metabolism and the biological oxidation pathways, with more than 60 out of 87 primary hits targeting each one of them (Fig. 2E).

As none of the three databases [71–73] had an updated ferroptosis-related pathway, we integrated a gene set curated from ferroptosis database, FerrDb [74] in both approaches. Based on the DrugBank interactions, none of the hits were related to any of the cell death models, as opposed to the text mining interactions, where ferroptosis and apoptosis were targeted by primary hits. Although the two cell death pathways were equally enriched, normalization with pathway sizes returned ferroptosis as more enriched than apoptosis (27% versus 18% of the pathway genes were targeted by primary hits) (Fig. 2E).

### Dose response curves and total axonal length analysis of primary hits

To assess further the potency and efficacy of primary hits in reversing the MCOS induced neurotoxicity, we performed a Dose Response Curve (DRC) and a retrospective Total Axonal Length analysis (TAL).

**Fig. 3:**
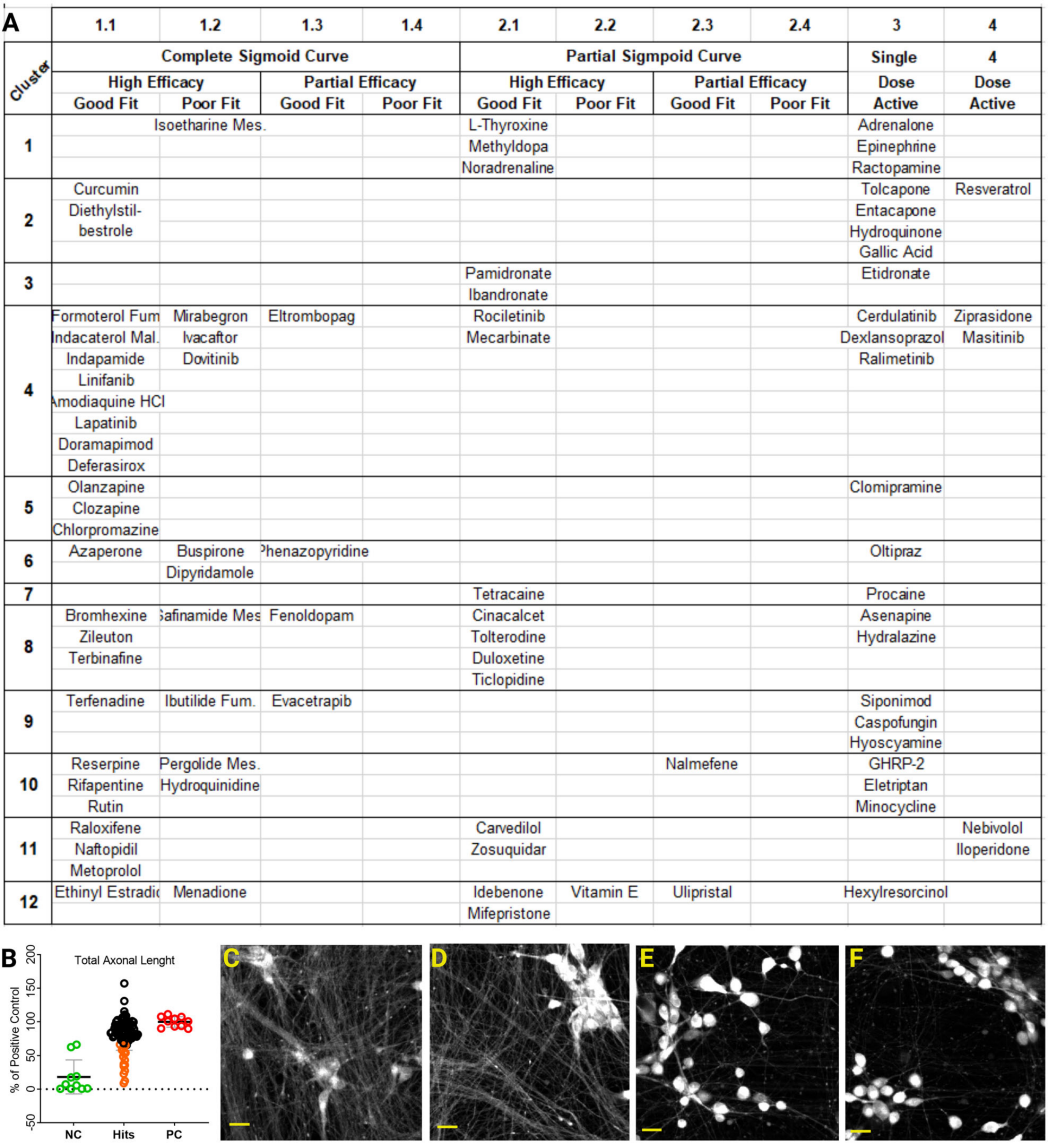
Secondary screen with DRC and TAL analysis. **A** Primary hits classified based on their dose response curves (DRC) asymptotes, their fit to the data (r2) and efficacies. 23 hist were excluded as single dose active molecules (group 3) together with two molecules that showed partial curve and efficacy (group 2.3). Five primary hits were active at all four highest concentrations (group 4). **B** Total axonal length (TAL) analysis of primary hits (black and orange) with positive (red) and negative (green) controls. *Y*-axis shows the %TAL of each hit versus the positive control. Primary hits that scored 2× standard deviations lower (orange) than positive controls were excluded. Each data point is the mean of *n* = 4 replicate plates. **C**, **D** Representative fields of positive control showing intact axonal network in comparison with Caspofungin-Acetate and Hyoscyamine (**E**, **F**), compounds with the lowest %TAL. Scale bar 20 µm.

Live neuronal counts of five concentrations from 5 μΜ to 8 nM, were fitted and plotted into groups per chemical cluster (see supplementary file and Fig. S3). The resulting DRC were classified based on their asymptotes, their fit to the data (r^2^), and their efficacies (Fig. 3A) according to previous studies [75, 76]. Twenty-three compounds were excluded as single dose (5 μM) active molecules and almost half of the primary hits showed complete curves and were further sub-classified based on their efficacies and their fit. Nineteen hits had partial curves, two of them with partial efficacies, were excluded from further validation.

Since axonal degeneration preceded the neuronal death in our MCOS phenotype, we explored whether primary hits retained live neuronal body counts but had degenerated axons. We performed a TAL analysis (Fig. 3B) by using the maximum projection of z-stack images from the 5 μM concentration of the DRC experiment. In total 19 small molecules (orange circles) of the primary hits (black circles) scored two standard deviations lower than the average of positive control (red circles) and were excluded from the hits list. Among them, we could not observe any enrichment for a specific chemical cluster, while six of them were excluded earlier as single dose active molecules in DRC analysis. Comparison of representative fields of positive control (Fig. 3C, D) versus molecules that failed TAL analysis, like Caspofungin Acetate (Fig. 3E) and Hyoscyamine (Fig. 3F), the differences in the axonal network were obvious.

### Influence of active hits on neuronal health

A secondary screen disclosed 49 active compounds that preserved intact axonal networks and prevented ferroptosis in a dose-dependent manner. It has been reported that OS causes damage to all macromolecules [77], thus, we investigated the health status of neurons after being rescued by these compounds. We quantitatively profiled multiple subcellular parameters of −5wAO depleted H1 neurons with the Cell Painting approach [78]. Features from plasma membrane, oxidized lipids, nuclei, mitochondria, lysosomes and microtubules of live neurons were used in dimensionality reduction with Principal Component Analysis (PCA).

**Fig. 4:**
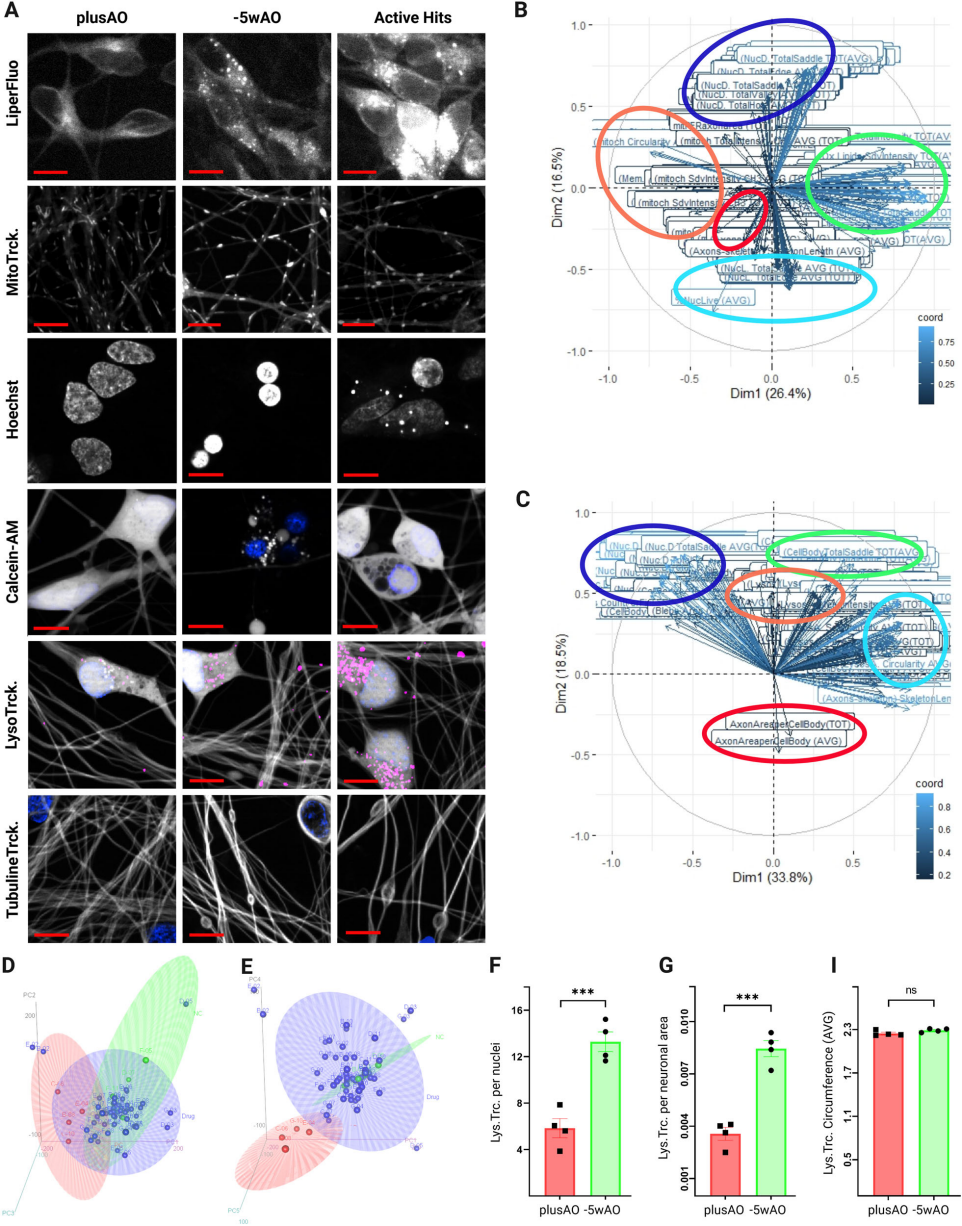
Active hits effects on neuronal health. **A** Representative fields of plusAO, –5wAO and active hits treated neurons. Fluorescence bio-probes (per lane) used in cell painting assay with *n* = 4 plates and 60× objectives. Scale bar 10 μm. **B**–**E** Principal Component analysis (PCA) of features extracted from image analysis (Cell PathFinder). **B**, **C** Graphs of variables for day8 and day9, showing features contributing to PC1 and PC2 (Dim1 and Dim2). **D**, **E** 3d animating plots of PC1-2-3 and PC1-4-5 for day8. Red and green are plusAO and −5wAO treated controls and blue the active hits. 90% confidence ellipses of Mahalanobis distances from centroids are shown. For fully animated graphs and day9 see vS05-vS08 videos. **F–I** LysoTracker™ targeted vesicle numbers in plusAO (red) and −5wAO (green) per nuclei (**F**) and per neuronal area (**G**) in MCOS treated day9 H1 neurons. No significant differences in the average lysosomal vesicle size (**I**). Data are mean, ± SEM of four technical repeats in *n* = 4 plates analyzed with unpaired *t*-test and *P* values 0.1234(ns), 0.0332(*), 0.0021(**), 0.0002(***), <0.0001(****).

The first five principal components (PC) explained more than 75% of the variation in our data set for each day. Graphs of variables (Fig. 4B, C) revealed an absolute clustering of features based on the phenotype, with the correlation in between them as expected. For example, a cluster of oxidized lipids (green) was the most contributing variable to PC1 (denoted as Dim1), while live-dead nuclei (dark-light blue) were in negative correlation and the second most contributing factors in variation (Dim2) (Fig. 4B). At day9 (Fig. 4C), with advanced neuronal death, live-dead nuclei clusters were the most contributing factors to PC1. Unexpectedly, day9 PC3 was defined by LysoTracker™ stained vesicles (orange). Comparison of controls with and without AO confirmed the difference in lysosomal numbers per nuclei (Fig. 4F) and neuronal area (Fig. 4G) with no differences in sizes based on the average circumference of vesicles (Fig. 4I). Although the exact mechanism is not yet clear, implication of lysosomes in ferroptosis has been reported earlier [79–81].

Animated 3d graphs of PC1-2-3 (Fig. 4D, vS05) and PC1-4-5 (Fig. 4E, vS06) for day8 and for day9 (vS07-vS08) neurons helped to exclude further nine small molecules from the active hits list. Representative fields of positive (plusAO) and negative (−5wAO) controls and the excluded active hits are shown in Fig. 4A.

### Differentially enriched pathways in H1 and H2 haplotypes under MCOS

**Fig. 5:**
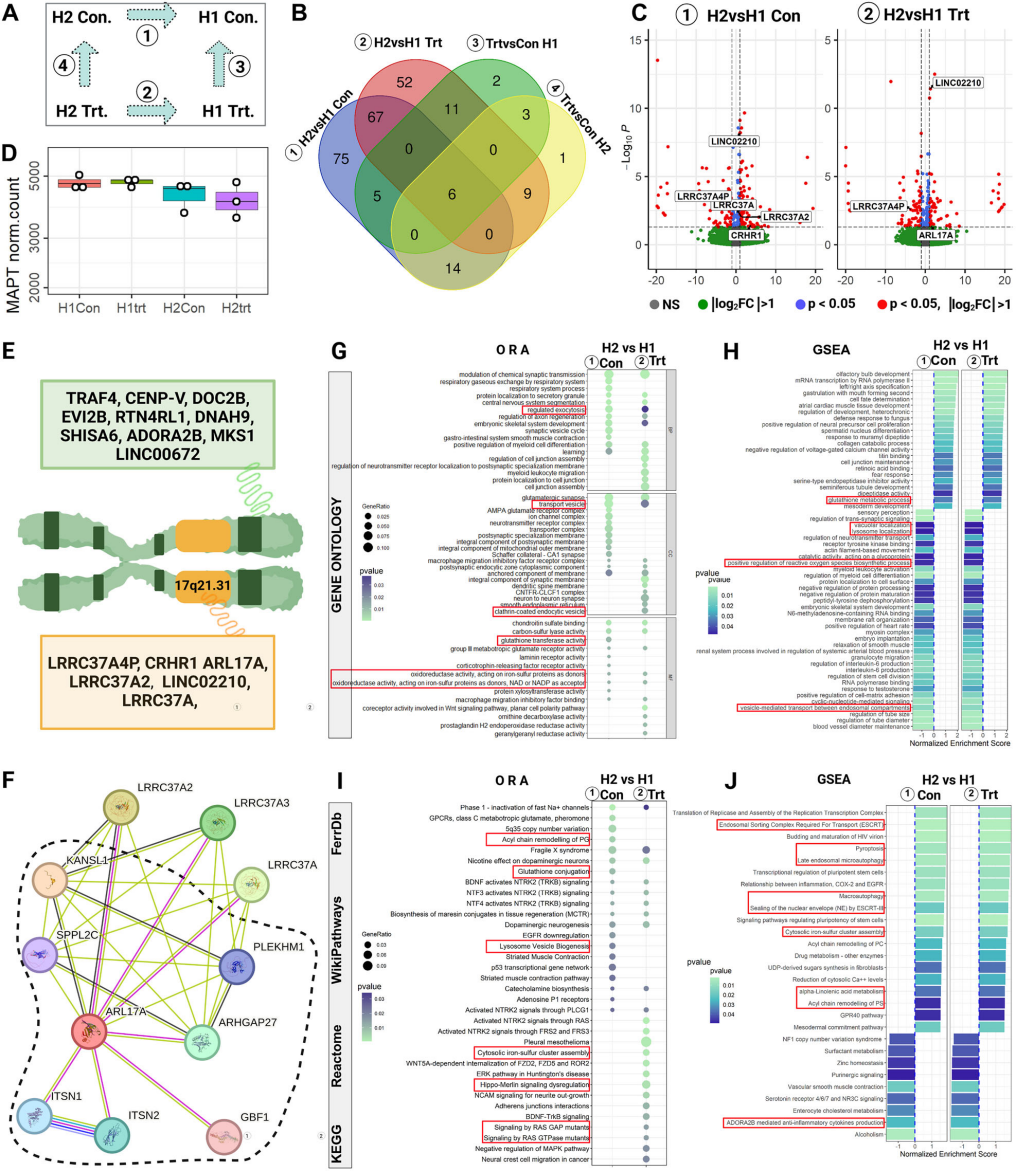
Transcriptional profile of H1-H2 haplotype neurons. **A** All four comparison groups (**①,** **②,** **③,** **④**) between haplotypes with plusAO (Con) and –5wAO (Trt). **B** Venn diagram with common and distinct DEGs between groups. **C** Volcano plots of DEGs with H2 versus H1 under **①** Cont (plusAO) and **②** Trt (–5wAO) conditions. On the *x*-axis the logFC and *y*-axis the -log10(*P*-value). Significant are DEGs (red dots) with *P*-values < 0.05, log_2_FC>|1|. **D** No difference in normalized MAPT transcripts in all groups (**①,** **②,** **③,** **④**). (**E**) All DEGs within the locus (yellow box) or outside of the locus, on chr.17 (green box). **F** *ARL17A* predicted interactor partners with 0.4 confidence (STRING) where line color indicates the type of interaction evidence, the thickness, and the strength of the data support. All interaction partners except GBF1, ITS1, and ITS2 are genes of chr:17q21.31 locus. All Interaction partners except LRCC37A genes are related to clathrin coated vesicles, endolysosomal vesicular fusion events and transferrin receptors (dashed line). **G–I** Overrepresentation analysis (ORA), **H–J** Gene Set Enrichment Analysis (GSEA) in Gene Ontology (upper panel) and in a custom list that covers KEGG, Reactome, WikiPathways, and FerrDb databases (lower panel). Red boxes denote ferroptosis and endolysosomal related pathways.

To assess the transcriptional signatures of haplotypes under MCOS, we performed Differential Expressed Genes (DEGs) analysis on day8 (axonal degeneration start) neurons treated with plusAO and −5wAO and compared all four groups (Fig. 5A).

The intra-individual (three per haplotype) variability was higher than the MCOS triggered variation for both haplotypes (groups ③ and ④, Fig. 5B). Whereas, in between haplotypes comparison of H2 versus H1 under ①Con and ②Trt conditions, returned approximately 150 DEGs for each group (Fig. 5B, C).

Copy number variations within the locus [1, 2, 82–84] intricates the complexity of the region, causing inconsistent DEGs in the literature [1, 2, 6, 24, 82, 83, 85, 86]. Our DEGs list included several genes within and outside of the locus, on chr:17 (Fig. 5E). The *LRRC37A4P* that is present only in H1 haplotype [2, 83], was significantly downregulated in H2 versus H1 in both conditions (① and ②). Interestingly, total MAPT was not a DEG (Fig. 5D) based on the SNP (rs242561) on Antioxidant Response Element [87], but in good agreement with protein levels determined in our previous study [35]. On the other hand, *CRHR1* expression was higher in H2, but AO depletion reduced its expression only in H2.

The ADP-Ribosylation Factor Like GTPase 17A (*ARL17A*) was significantly higher only in H2 versus H1 Trt group ②, that showed resistance to MCOS earlier (Fig. 5C). *ARL17A* has unknown functions, but it is a member of the ADP-ribosylation factor (ARF)/ARF-like protein (ARL) family [88]. *ARL17A* predicted interactors are mainly genes within the locus (Fig. 5F), while almost all of its partners (dashed lines) are directly or indirectly involved in vesicle trafficking, the endolysosomal system, and transferrin receptors [89].

ORA among DEGs (Fig. 5G–I) and Gene Sets Enrichment Analysis (GSEA) (Fig. 5H–J) with Gene Ontology (Fig. 5G, H) and a custom list including “KEGG”, “Reactome”, “WikiPathways”, and ferroptosis Database “FerrDb” (Fig. 5I, J), showed the enriched pathways that emerged from H2 versus H1 haplotypes under Con and Trt conditions (①-②). Comparison of clusters returned the Glutathione metabolic processes, the ROS activity on iron sulfur proteins, the acyl chain-lipid metabolism, and the Hippo-Merlin pathways, all related to ferroptosis [90], as differentially enriched pathways. Other enriched pathways like clathrin coated endocytic vesicles, lysosomal biogenesis, and localization confirmed our previous results with Cell Painting assay for lysosomal implication.

### MCOS induced ROS and Lysosomal dynamics in H1-H2 haplotypes

**Fig. 6:**
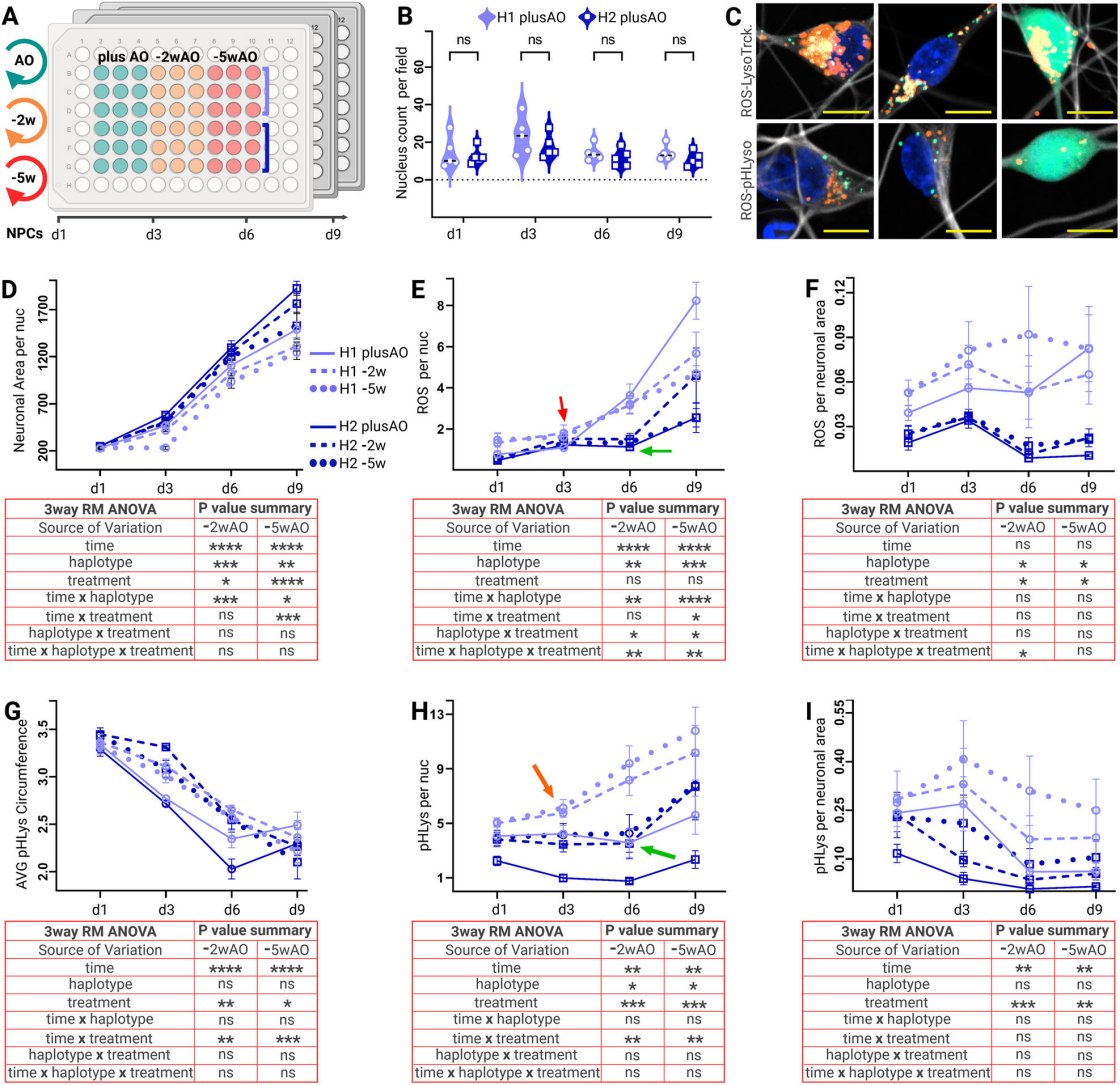
Differential ROS and Lysosomal dynamics in H1-H2 haplotypes during neuronal differentiation. **A** Longitudinal assay schema evaluating the interaction of genetic background (H1 versus H2) with MCOS (plusAO, −2wAO, −5wAO) over time (days 1,3,6,9). Data are mean, ± SEM of three biological replicates per haplotype (rows B, C, D light blue for H1 and E, F, G dark blue for H2) with three technical and five experimental repeats, each lasting ~7 weeks. **B** No significant differences between haplotypes on live nuclei numbers with plusAO on each day of neuronal maturation with unpaired *t*-test, *p* < 0.05. **C** Representative fields of H1 (left and right) and H2 (middle column) neurons with ROS (green), LysoTracker-Red (upper row), and pHLys-Red (lower row). **D–I** Three way Repeated Measures (RM) ANOVA of plusAO (solid lines), −2wAO (dashed), and −5wAO (dotty) treated cells. H1 (light blue) and H2 (dark blue) haplotypes. *P*-values summaries in the tables below indicate 0.1234 (ns), 0.0332 (*), 0.0021 (**), 0.0002 (***), 0.0001 (****). **D** Average neuronal area per nuclei. ROS dots accumulation over time per nuclei (**E**) and the neuronal area (**F**). Red and green arrows indicate the differential time of ROS accumulation for haplotypes (d3 for H1 and d6 for H2). **G** Average lysosomal circumference for both haplotypes. Lysosomal counts per nuclei (**H**) and neuronal area (**I**). Red and green arrows indicate the differential time of lysosomal increase per haplotype (d3 for H1 and d6 for H2).

After cell painting assay and the enriched pathways results, we studied the ROS accumulation and the Lysosomal dynamics in a longitudinal experiment of both haplotypes treated with plusAO, −2wAO, and −5wAO, during neuronal maturation stages (d1, d3, d6, d9) (Fig. 6A).

ROS (green) appeared as a dotty signal (Fig. 6C left and middle) throughout the maturation stages. At d9 (the average H1 death initiation) a more diffuse ROS signal was observed (Fig. 6C right column). Initial co-staining of ROS with LysoTracker™ Red DND99 (Invitrogen), that targets a plethora of acidic vesicles other than lysosomes, showed very high levels of overlapped signal for both haplotypes (Fig. 6C upper row), whereas co-staining with the more specific lysosomal marker pHLys-Red (Dojindo, Japan) showed no overlapping (Fig. 6C lower row).

A parallel study by Yan et al. [91], showed that lower cell densities are more prone to ferroptosis. Thus, we compared live nuclei counts in plusAO treated H1 versus H2 over time (Fig. 6B) and in plusAO versus −2wAO and −5wAO treated H1 and H2 haplotypes (not shown) the next day of seeding (d1). No significant differences in seeded neuronal densities were observed. In contrast, the neuronal area (size) per nuclei between treatments and the haplotypes over time (Fig. 6D) returned time as the most and the haplotype as the second most significant factors of variation. The treatment was more significant with −5wAO than the −2wAO (also evident in the two-level interactions of time X treatment) in good agreement with the chronic OS model. The slight reduction in significance for haplotype alone or in combination with time X haplotype is probably due to some toxicity in H2 lines, a phenomenon observed earlier in cytotoxicity assay as well.

In comparison of ROS signal per nuclei (Fig. 6E), time was the most and haplotype the second most significant source of variation. While the treatment was significant only in combination with haplotype or time at −5wAO depletion, in alignment again with the mild chronic OS model. The differential time of neuronal maturation for ROS acceleration, that was d3 for the H1 (red arrow) and d6 for the H2 (green arrow) haplotype, in the graph of data, confirmed our cytotoxicity results. At d9, that is the average H1 death initiation, the ROS signal per nucleus became slightly unstable due to nucleus debris in the DAPI channel of AO depleted cells. The same ROS signal expressed per neuronal area (Fig. 6F), which increased over time (Fig. 6D), as expected showed no variance in time. The haplotype and the treatment were still significant but with lower *p*-values due to high variability in H1 as seen in the graph Moreover, ROS accumulation in H2 haplotype was directly proportional to neuronal area with the exception of a small dip after d3 due to rapid increase in neuronal size after addition of neurotrophic factors (BDNF, GDNF, NT3) (See material and methods). Contrarily, the H1 haplotype had more ROS in overall conditions, followed an increasing pattern over time and was more responsive to −2wAO and −5wAO depletion than the H2.

Analysis of Lysosomal dynamics with the specific pHLys-Red marker returned no haplotypic difference on average lysosomal size, despite the strong reduction over time and a small increase due to AO depletion (Fig. 6G). Whereas, −2wAO and −5wAO treatment increased significantly lysosomal numbers per nuclei (Fig. 6H) for both haplotypes. This increase in numbers over time, although it followed almost a similar pattern for both, was higher in H1 than the H2 haplotype for all conditions. The bending time point of rapid lysosomal biogenesis, similarly to the ROS signal, was earlier for H1 (d3, red arrow) than the H2 (d6, green arrow) haplotype. Finally, lysosomal numbers per neuronal area increased significantly after AO depletion and decreased over time equally for both haplotypes (Fig. 6I).

## Discussion

NDs are the second leading cause of death worldwide, affecting 15% of the global population and expected to double in the next decades [92]. Hence, there is tremendous effort to untangle the interplay of genetic risk factors with the environmental parameters in the etiology of progressive neuronal deterioration, a common hallmark of several NDs.

GWAS have consistently identified the chr:17q21.31 locus H1 haplotype as a major risk factor for a wide spectrum of NDs, including tauopathies, synucleinopathies, ET, and ALS [8–13, 15–19], indicating a common pathological signature, rather than a ND type-specific pathway. Yet, the complex genomic structure of the region [1, 3, 82] has so far precluded the identification of causal gene(s) or variant(s) responsible for the risk association.

Here, we performed a combinatory study of genetic variation (H1 versus H2), within the context of OS, another major contributor of NDs [25, 77, 93]. Instead of chemical treatment, we triggered OS with AO depletion for ~1.5 months, as a more relevant model to the pathophysiology of NDs. Induction of MCOS led us to the identification for the first time, of a significant haplotype-specific difference in susceptibility that might have gone unnoticed after harsh OS conditions. The increased sensitivity of H1 haplotype to MCOS could contribute to the association with NDs. Furthermore, the utilization of iPSCs was essential for the identification of this phenotypic difference, since derivation of H2 haplotype from Homo Neanderthalensis [94] excludes the applicability of this phenotypic difference on non human model organisms.

Blebs or swellings are considered early pathological features [95, 96] of degenerating axons after different perturbations, including OS [28–30, 59]. However, transient axonal swellings occur also in healthy neurons, either during big cargo transport movements [97], or during action potential enhancement [98]. We showed on live neurons that the MCOS induced blebs on the sensitive H1 haplotype were regions of disorganized microtubule bundles that increased in size and numbers on progressively thinning and degenerating axons before the cellular death. The above-mentioned blebs, other than some transient elongated swellings due to big cargo movements, were absent in AO depleted H2 or plusAO treated neurons in parallel. Considering that several FDA-approved drugs prevented the neuronal death but not the axonal degradation, an interesting future direction of investigation would be whether these two outcomes are connected or independent parallel events.

Several lines of evidence in the current study pointed to ferroptosis as the neuronal death mechanism induced by MCOS treatment. Although it is a recently described cell death mechanism, ferroptosis characteristic features of iron implication and lipid peroxidation in postmortem brains of patients with NDs have been described long before [99]. We identified a considerable number of ferroptosis inhibitors as primary hits, while apoptosis and autophagy inhibitors failed to rescue the phenotype in our screens. Ferroptosis pathway was enriched in the text mining approach of hits-proteins targets, while a two-fold increment in ferroptosis inhibitors was achieved at the end of the screening assays and hits validation. Additionally, lipid peroxidation a biochemical hallmark of ferroptosis [100] that is evident in numerous NDs [101, 102], was the first principal component defining our MCOS phenotype in cell painting assay. The spreading and the propagation of axonal degeneration and the neuronal death in a wave-like manner in time lapse images was reported earlier as a ferroptosis marker [60–62]. Finally, ferroptosis related pathways like glutathione metabolic processes, ROS activity on iron-sulfur proteins, and lipid metabolism [90] were differentially enriched pathways in H2 versus H1 comparison under basal and MCOS conditions.

In the MCOS induced phenotype, despite the prolonged time of AO depletion, NPCs and immature neurons of both haplotypes were unaffected, until they developed complex axonal networks. This is in good agreement with the pleiotropic nature of ROS as crucial molecules in maintenance and lineage specification of neural stem cells [103–105], but also being toxic with the vulnerability depending on the neuronal type and the maturation stages [106, 107]. Monitoring neuronal maturation under MCOS and plusAO conditions in a longitudinal assay showed initially low levels of ROS for both haplotypes. Induction of differentiation with neurotrophic factors caused a rapid increase in ROS levels for all conditions, indicative of the metabolic shift to oxidative phosphorylation [108, 109]. However, the average increase in ROS levels per neuron started earlier for the H1 than the H2 haplotype. Moreover, ROS levels were directly proportional to the neuronal area/size only in the H2 haplotype. Whilst in H1 lines the ROS to neuronal size ratio showed a cumulatively increasing effect over time and prolonged weeks of AO depletion, in alignment with the haplotypic differences observed in neurotoxicity assay. The comparatively elevated ROS levels that triggered an earlier ferroptotic death in H1 versus the H2 lines, could be a result of either increased ROS production in H1 or more efficient elimination in H2 haplotype.

Incomplete autophagy-lysosomal clearance pathways and protein aggregation are common features for NDs [110–113], while recent studies revealed the strong link between lysosomes and ferroptosis [81, 114, 115]. Lysosomes are dynamic organelles that respond to stimuli by regulating their numbers, sizes, and localization [116]. They are involved in vital intracellular functions like degradation, membrane repair, phagocytosis, endo/exocytosis, and nutrient sensing [117]. In cell painting and the longitudinal assays, we showed that MCOS induced an increase in lysosomal numbers for both haplotypes. Similarly, an increase in lysosomal numbers was reported after H_2_O_2_ mediated sub-lethal OS induction on myoblasts, which incidentally did not induce apoptosis and was reversed by iron chelators, implying ferroptosis activation [118].

Comparison of DEGs between the haplotypes returned the small GTPase, *ARL17A* as a differentially expressed gene in the resistant H2 allele under MCOS. Family members of small GTPase are well documented modulators of Lysosomal mediated plasma membrane repair, the vesicular biogenesis, transport, and fusion events [119, 120]. Related pathways like clathrin mediated endocytosis, the endolysosomal system, and the vesicle transport, were enriched after the analysis either with primary hits-protein targets obtained from Drugbank, or the RNA-sequencing. Identical biological functions were proven earlier for the predicted interactor partners of *ARL17A*, located mainly within the haplotype region. Based on the above and considering the function of other locus genes, we propose that chr.17q21.31 is a genomic region of functionally related genes cluster [121], implicated in the endolysosomal system. In a parallel study [84] the SNARE signaling, that mediates vesicular fusions with target membranes, was enriched in Ingenuity Pathway Analysis with chr:17q21.31 locus genes. In addition, a recent GWAS of pleiotropy between three major NDs identified the chr:17q21.31 as the only shared risk locus among AD, PD, and ALS, implicating it also in lysosomal-autophagy dysfunction and oxidative stress. [122]. Here, we provide experimental evidence linking the H1 risk haplotype with the endolysosomal system, in line with the defective vesicular functioning commonly observed in proteinopathy driven NDs [123].

The above hypothesis was in good alignment with the differential lysosomal dynamics we observed between the two haplotypes. The comparatively lower lysosomal numbers in H2 could be due to *ARL17A* mediated increased plasma membrane fusion and exocytosis events, according to a recently published anti-ferroptotic mechanism of lysosomal release of oxidized lipids and proteins [114]. In parallel, transferrin receptor trafficking from membrane to the endolysosomal system by small GTPase family members, is already documented [124, 125]. *ARL17A* mediated reduction in transferrin receptors, and consequently iron uptake, might prevent ferroptosis in H2 haplotype under MCOS conditions. Alternatively, the higher lysosomal load in H1 cell lines could make them more prone to ferroptosis than H2, since lysosomal retention of cystine in cancer cells decreased cytoplasmic cysteine and glutathione (GSH) levels [115]. Lower levels of the major ferroptosis inhibitor and antioxidant molecule GSH [126], could explain the earlier and the rapid increase in ROS signal observed in H1 haplotype. More detailed mechanistic work needs to be done for the clarification of the differential lysosomal load between the two haplotypes and its impact on ferroptosis susceptibility.

The translation of chr:17q21.31 genetic associations with NDs to empirical evidence has failed so far for many reasons. Polygenic prediction models showed that complex diseases are driven by several minor effect size causal variants in a gene to gene and gene to environment interaction model. Here, we showed for the first time the phenotypic difference that is attributable to SNPs and variants in linkage disequilibrium, on functionally related genes clustered within a defined genomic locus. The altered endolysosomal system in H1 versus H2 haplotype, made neurons more susceptible to mild OS conditions even in healthy donors. It is anticipated that co-occurrence of H1 haplotype with other NDs related variants, causing cumulative protein misfolding and aggregations or OS, will exaggerate the causality of risk genes. Consideration of this haplotype-specific difference in future studies, preclinical evaluations, and diagnostics might advance precision medicine and disease management in NDs.

### Reporting summary

Further information on research design is available in the Nature Research Reporting Summary linked to this article.

## Supplementary information

Supplementary Information

Reporting Summary

vS01_H1 diku treated with plusAO corresp. Fig.1E

vS02_H1 diku treated -5wAO corresp. Fig.1F

vS03_H2 uilk treated with plusAO corresp. Fig.1G

vS04_H2 uilk treated -5wAO corresp. Fig.1H

vS05_3dplotPC1-2-3d8CP90 Ellips_movie corresp. Fig.4D

vS06_3dplotPC1-4-5d8CP90 Ellips_movie corresp. Fig.4E

vS07_3dplotPC1-2-3-d9CP90 Ellips_movie no corresp. Fig

vS08_3dplotPC1-4-5d9CP90 Ellips_movie no corresp. Fig

vS09_diku: H1 corresp. Fig.S1H -5wAO day9

vS10_yemz: H1 corresp. Fig.S1I -5wAO day8

vS11_lepk: H1 corresp. Fig.S1J -5wAO day9

vS12_qolg: H2 corresp. Fig.S1K -5wAO day12

vS13_zihe: H2 corresp. Fig.S1L -5wAO day12

vS14_uilk: H2 corresp. Fig.S1M -5wAO day21

## Data Availability

Raw RNA-seq data are submitted to the Sequence Read Archive (SRA) of National Institutes of Health (NIH) (https://www.ncbi.nlm.nih.gov/sra/PRJNA1258758). This paper does not report original code. Microscopy data reported in this paper will be shared by the lead contact upon request. Any additional information required to reanalyze the data reported in this paper is available from the lead contact upon request.
