# Supplementary material for: Chr:17q21.31 locus risk haplotype H1 susceptibility to ferroptosis is mediated by endolysosomal pathway
Source: Cell Death Dis. 2025 Nov 13;16(1):828. doi: 10.1038/s41419-025-08147-1 (PMC12618564; doi:10.1038/s41419-025-08147-1)
Supplement: Supplementary file 1 — Supplementary Information [file 41419_2025_8147_MOESM1_ESM.docx]

**Chr:17q21.31 locus risk haplotype H1 susceptibility to ferroptosis is mediated by endolysosomal pathway.**

Eldem Sadikoglou^1🖂^, Daniel Domingo-Fernández^2^, Natalia Savytska^1^, Noemia Fernandes^1^, Patrizia Rizzu^1^, Anastasia Illarionova^1^, Tabea Strauß^3,4^, Sigrid C. Schwarz^3,5^, Alpha Kodamullil^2^, Günter U. Höglinger^3,6^, Ashutosh Dhingra^1^, Thomas Gasser^1,7^, Peter Heutink^1^

**Supplementary to Neurotoxicity and death propagation, Figure 1**

Nine NPCs, three per haplotype (the H1, the H2 and the heterozygous H1/H2) derived from iPSCs of healthy donors (see material and methods), were treated and differentiated into neurons under plusAO, -3wAO, -4wAO and -6wAO depletion.

Relative fluorescence units were determined after Calsein-AM treatment for 30 min at 37^o^C. Comparison of each cell line with (solid bars) and without AO (stripped bars) on day10 and days12/13 of neuronal maturation showed a clear difference in viability between the haplotypes (**Fig.S1A**). H1 neurons were more susceptible to MCOS than the H2, while the heterozygous H1/H2 showed intermediate sensitivity. The H2 line, zihe at -4wAO and -6wAO showed even higher live neuronal counts than the plusAO treatment (significance in gray). Moreover, an additive effect of neurotoxicity was detected in all cell lines by increasing days of neuronal maturation (day10 versus days 12/13) and increasing weeks of AO depletion.

At the NPC stage (**Fig.S1B-C**) we did not observe any differences between haplotypes or between treatments, even after prolonged time periods of MCOS conditions (up to 10wAO depletion was tested).

In -5wAO treated day8, H1 neuron blebs and axonal degradation appeared initially at sub-regions of wells and propagated to adjacent regions while the neuronal cell bodies adapted a more round and swollen shape before their death (**Fig.S1E**). The -5wAO H2 haplotype cell lines (**Fig.S1G**) and the plusAO H1 and H2 cell lines treated in parallel


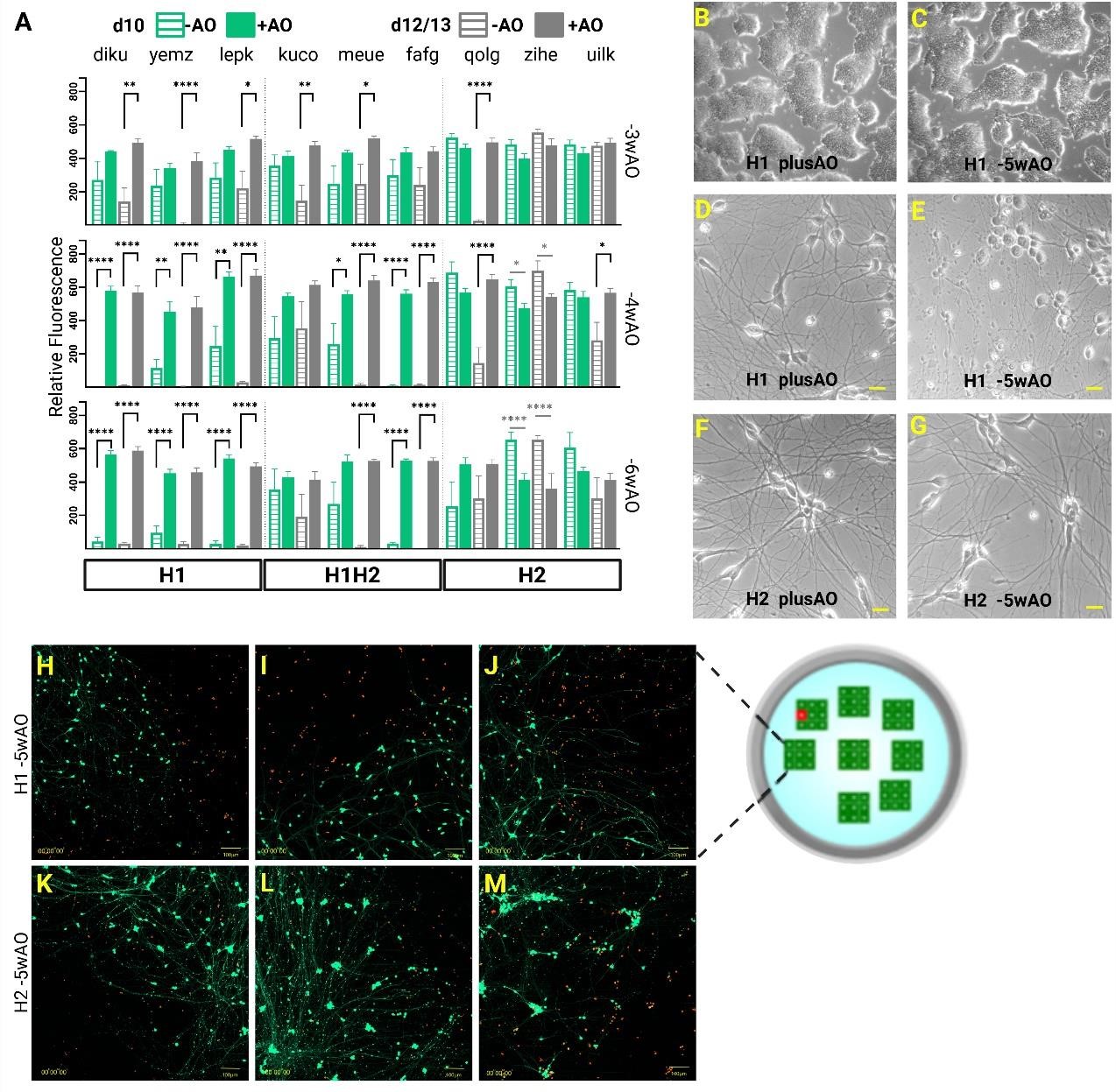


**Fig.S1: Differential susceptibility of H1-H2 haplotypes to MCOS induced axonal degeneration and neuronal death.** (**A**) Cytotoxicity assay with three cell lines (names in the upper panel) per haplotype, the H1, H2 and the heterozygous H1/H2. NPCs were treated and differentiated to neurons with (solid bars) and without AO (stripped bars) for indicated weeks. Relative fluorescence units of Calcein-AM on day10 (green bars) and days12/13 (gray bars) of neuronal maturation. Unpaired t-test comparison of plus to minus AO treated cell lines from two independent experimental replicates. Values are mean and ±SEM. P-values are 0.1234 (ns), 0.0332(*), 0.0021(**), 0.0002(***). (**B-C**) Representative bright field images from H1 cell line (diku) NPCs treated with plusAO (B) and -5wAO (C). (**D** to **G**) Differences of axonal degeneration and neuronal death in representative bright field images from day8 neurons of H1 (diku, D,E) and H2 (uilk, F,G) with plusAO (left column) and -5wAO (right column). Scale bar 100 pixels on ImageJ. (**H** to **M**) Representative fields of neuronal death spreading in population. Time lapse for 24h (1fph) of tiled images from 3X3 fields and DEAD\LIVE kit (red/green). Upper row H1 (diku, yemz, lepk), lower row H2 haplotype (qolg, zihe, uilk) cells at -5wAO depletion. H-I imaged on day9, J-K-L day12 and M day21 of neuronal differentiation. Scale bar 100μm. For complete time-lapse movies refer to supplementary videos vS09 to vS14 respectively.

25

(**Fig.S1D-F**) retained intact axonal networks. To monitor the spatiotemporal progression of axonal deterioration we treated all cell lines of H1 and H2 haplotypes with -5wAO depletion and stained with LIVE/DEAD kit (green/red) on days 9, 12 and 21 of neuronal maturation. In time-lapse images for 24h of 3X3 tiled fields and several regions within the wells, we recorded the propagation of neuronal death in a wave-like manner in all cell lines (**Fig.S1 H-I-J-K-L-M**). The difference between the haplotypes was the day of maturation at which the neuronal death initiated, as it was earlier for H1 than in H2 cell lines in good agreement with cytotoxicity assay.

**Supplementary to Primary Screen, Figure 2**

**FDA approved chemical-library screening assay**

In order to establish the screening assay conditions, we specified several parameters. For the screening purposes we used live neuronal counts after Calcein-AM (red) staining and high content microscopy on -5wAO treated H1 neurons (diku) (**Fig.S1A**). The rescue was tested with complete or diluted AO supplementation on day3 and day6 of neuronal maturation. Four different time points (d8, d10, d2, d14) of imaging and two concentrations of solvent Dimethyl sulfoxide (DMSO) were tested. Results are the mean and ±SEM from three experimental repeats analyzed with One-Way ANOVA and Sidak`s Post Hoc test.

Day6 was selected as the latest day in reversing the neurotoxicity by AO supplementation, since there was no significant difference in survival rates between day3 and day6 rescued neurons at all four time points (**Fig.S2B**). For the tested imaging time points (days 8, 10, 12, 14) the latest optimal time was determined to be day12 of neuronal maturation, where the difference in live neurons between day6 rescued versus not rescued was significantly the highest (**Fig.S2B**). Imaging at day14 was excluded as some neurotoxicity was observed in overall conditions due to nutrients restrictions after exceeding one week of media change intervals.

The observed neurotoxicity was specific to AO depletion in a time and dose dependent manner. Supplementation of media with full and 1/25 diluted AO on day6 could reverse the neurotoxicity completely in all four time points. On the contrary the 1/625 dilution of AO could reverse the neurotoxicity completely on day8, only in half of the neurons on days 10 and 12 and not on day14 (**Fig.S2B**).

Another major parameter that had to be specified was the DMSO concentration. DMSO was the solvent of FDA-approved chemicals library, which is known to be toxic on primary neurons [127]. To determine the highest applicable drug concentration we checked the highest tolerable DMSO concentration by neurons under MCOS. We rescued the neurotoxicity on day6 as before and tested 0%, 0.5% and 1% concentrations of DMSO (**Fig.S2C**). The 0.5% of DMSO was the highest tolerable concentration since 1% DMSO caused additional toxicity on days 10, 12 and 14 of neuronal maturation (**Fig.S2C**).

The chemicals library (Selleckchem) was aliquoted across 30 deep well plates (named HTS1 to HTS29, **Fig.S2D**) including 10 control wells per plate and was repeated in n=4 times. The screening assay quality was determined with z-factor [128] per plate based on the controls on average at z-factor=0.87 and z-factor=0.68 for the two batches respectively (**Fig.S2D**). The correlation of replicate plates (**Fig.S2E**) was determined on average at R^2^=0.85 with HitSeekR [43].


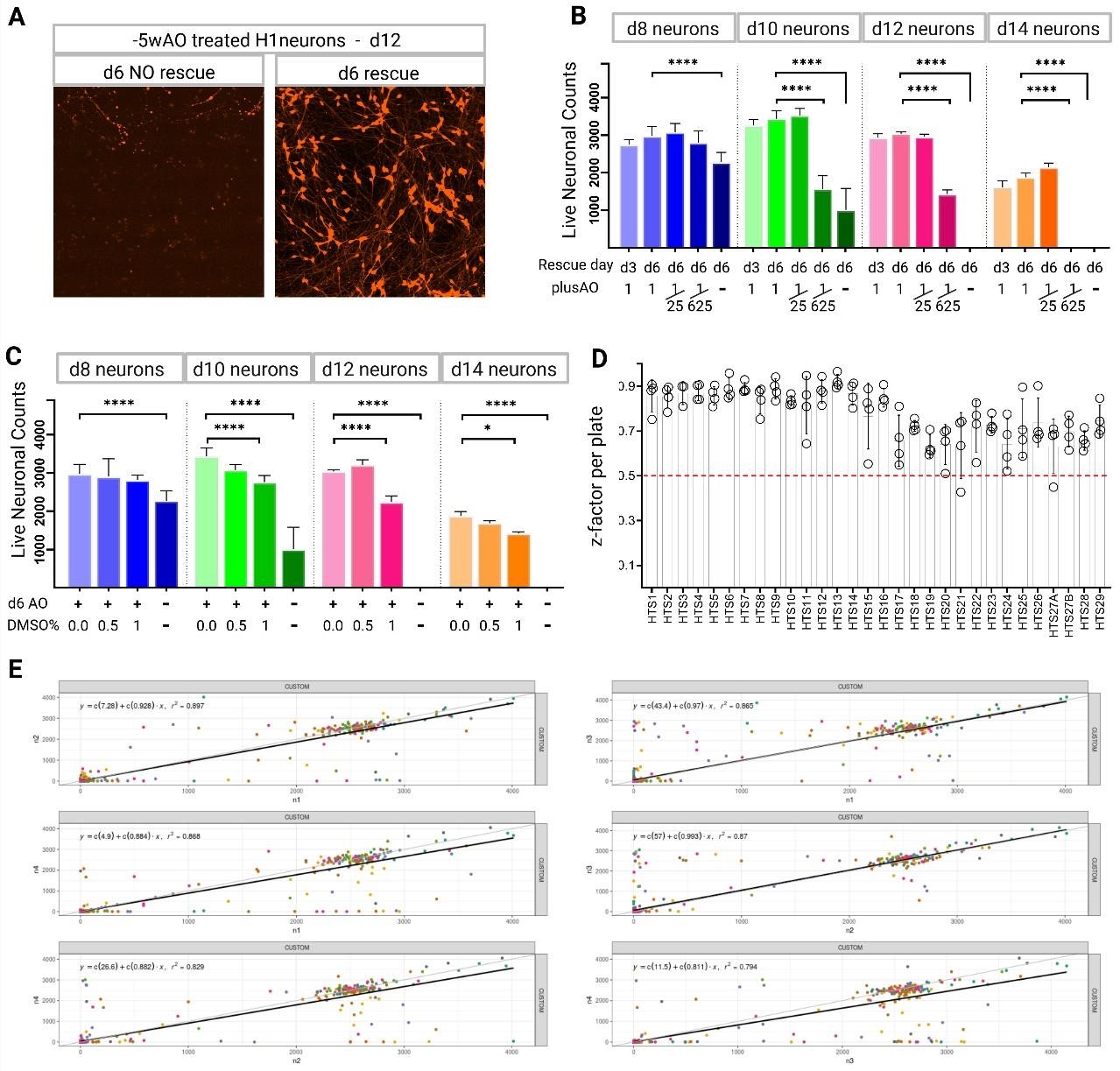


**Fig.S2: FDA-approved chemical library screening with MCOS sensitive H1 neurons.** (**A**) Representative field of day12 neurons imaged with Calcein-AM (Red) of negative (no rescue on day6) and positive (day6 rescue) controls. (**B**) Live neuronal counts on days 8, 10, 12 and 14 of neuronal maturation from -5wAO treated H1 neurons (diku) and rescued with AO supplementation on day3 and day6. Rescue with full AO and diluted to 1/25 and 1/625 on day6 were tested in three experimental replicates. Results are the mean and ± SEM compared with one-way ANOVA and Sidak's post Hoc test. (**C**) The maximum DMSO concentration which is tolerable by H1 neurons under MCOS on day6 rescue was determined to be the 0.5%. Since the 1% of DMSO is significantly different from no DMSO added on day6 rescue. Results are the mean and ± SEM compared with one-way ANOVA and Sidak's post Hoc test. P-values are 0.1234 (ns), 0.0332(*), 0.0021(**), 0.0002(***). (**D**) Primary screening assay quality measurement with plate wise z-factors from n=4 replicates per screening plate (HST1 to HTS29) based on positive and negative controls. (**E**) n=4 replicates correlation plot including a linear regression (black line) with corresponding R2 correlation factor from primary screen analysis with HitSeekR.

**Supplementary to Primary Screen, Figure 2**

**Chemical structure similarity analysis of primary hits, Table S1-S2**

Since, cellular AO defense systems is a complex, multi-molecular regulatory pathway with enzymatic and non-enzymatic inhibitors, cofactors, scavengers and metal chelators [129] it was not surprising that we obtained a quite functionally and structurally diverse groups of molecules as primary hits. Structure similarity analysis returned twelve clusters of primary hits (**Table S1-S2**). Setting up the threshold lower to obtain more clusters was not necessary as it would separate the Gallic acid and the Hydroquinone from Cluster 2 into an independent cluster (Table S1 Cluster 2 blue dashed box). On the other hand, setting the threshold higher to obtain less clusters would merge Clusters 9 and 10 to a more structurally diverse and complex group. For comparison we included chemical molecular targets given from the supplier (Selleckchem), their indications from Drugbank (https://go.drugbank.com/) and their classification according to Chemical Entities of Biological Interest (ChEBI) ontology (Jan, 2022).

Various known AO were spread across different clusters. Vitamins E and K were grouped in Clusters 12. Polyphenolic compounds like phenylalanine derivatives, catecholamines in Cluster 1, stilbenes and flavonoids in Cluster 2. Cluster 4 which was the biggest and the most diverse group had antineoplastic and anti-inflammatory drugs. All the tyrosine kinase inhibitors, together with quinoles, and indoles consisted mostly of the group. Small clusters like 3, 5, and 7 contained functionally and structurally related drugs with similar therapeutic uses. For example bisphosphonates of Cluster 3 that are indicated for bone disease treatment [130], were reported to retain AO properties by either inhibiting the lipid peroxidation and the Fenton reaction and/or due to their

96 calcium chelating activity [131].

**Table S1: Chemical structure classification of primary hits.** Clusters 1 to 7 after Exact SAHN clustering of primary hits based on their DayLight fingerprints and Ward's linkage method (Scaffold Hunter v2.6.3). Chemical structures of primary hits based on Chemical Entities of Biological Interest (ChEBI) ontology (May, 2022) are shown. Molecular targets and drug indications given by Selleckhem with classifications from ChEBI ontology (Jan, 2022).


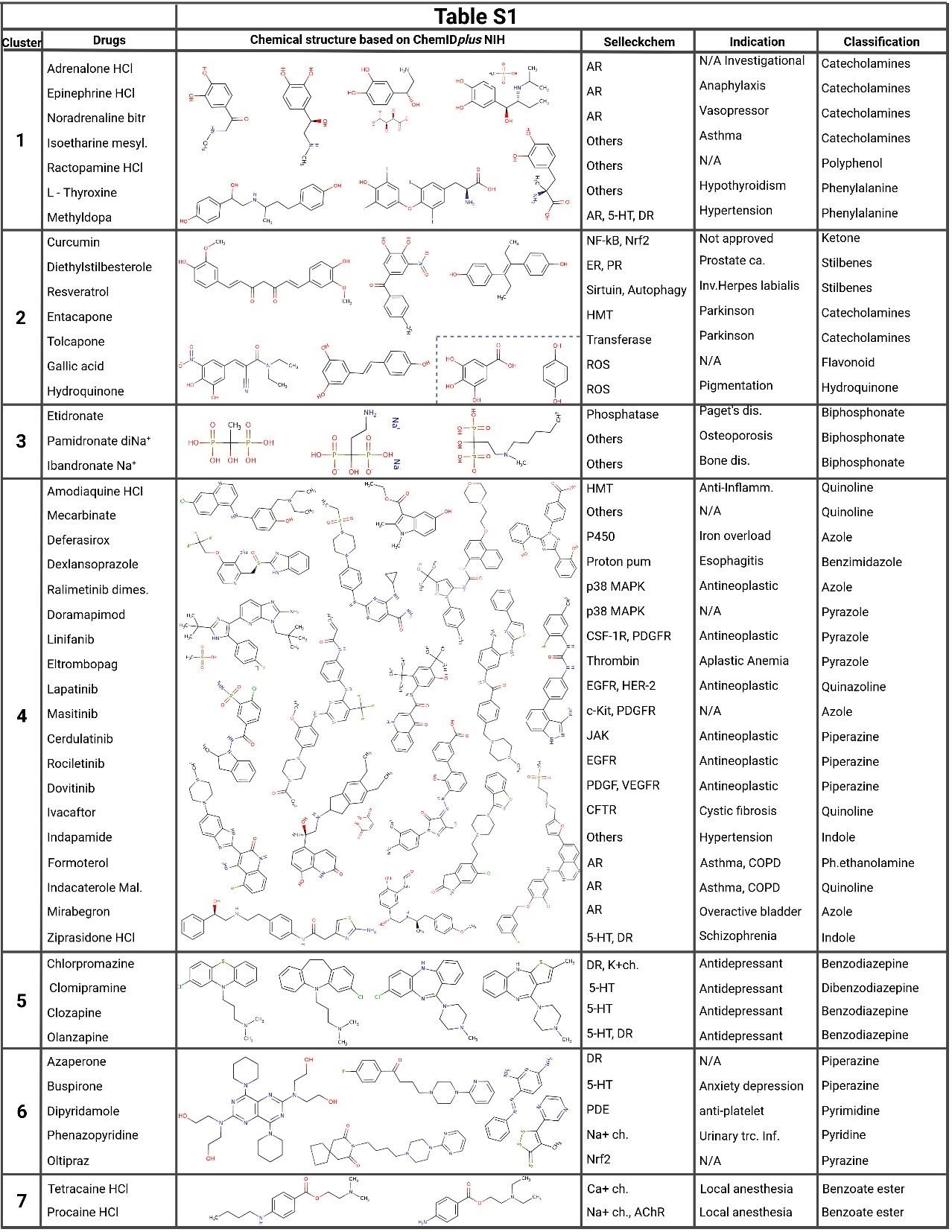


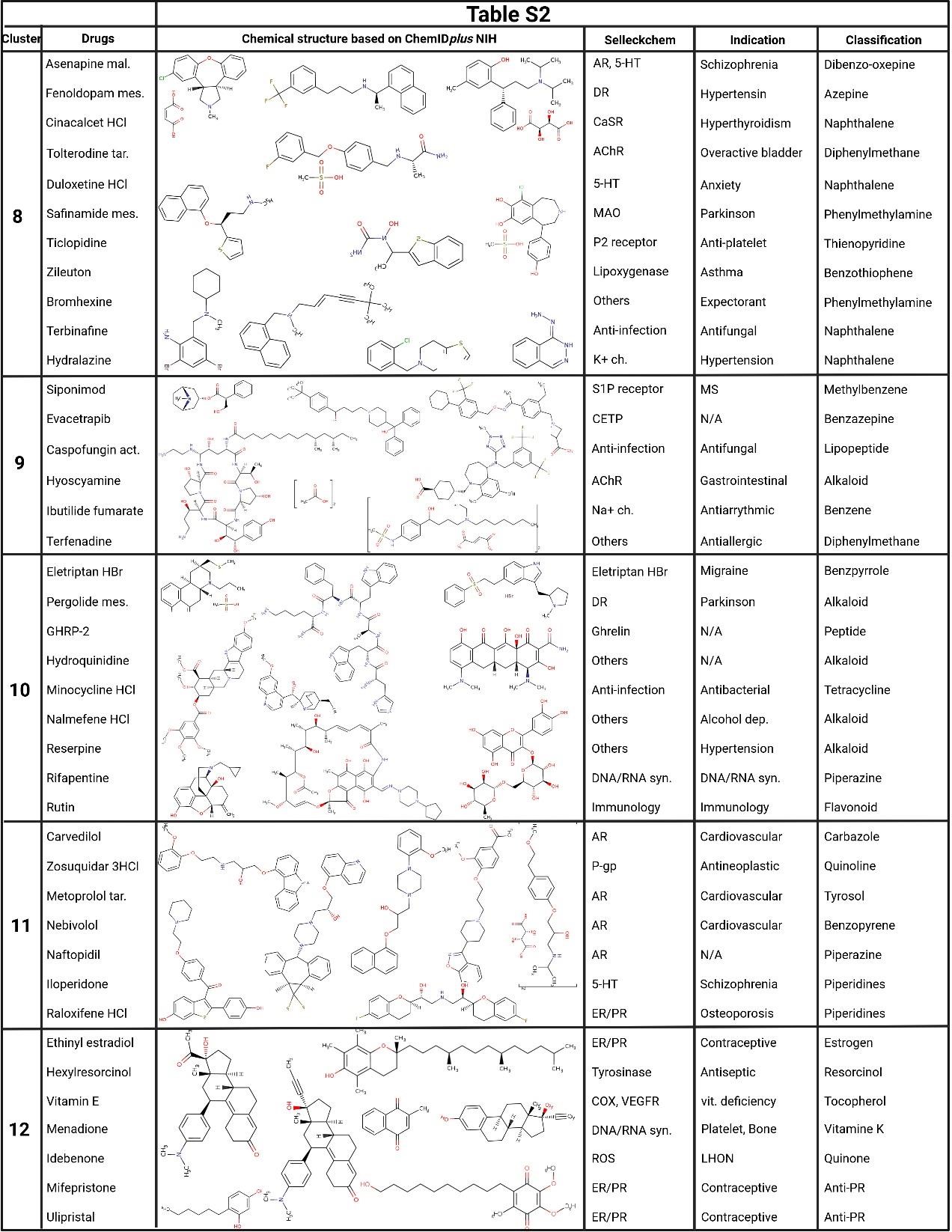


**Table S2: Chemical structure classification of primary hits.** Clusters 8 to 12 of primary hits based on their DayLight fingerprints (Scaffold Hunter v2.6.3). Chemical structures from Chemical Entities of Biological Interest (ChEBI) ontology (May, 2022), molecular targets and drug indications given by Selleckhem and classifications from ChEBI ontology (Jan, 2022).

**Supplementary to Figure 3**

**Secondary Screen**

In the next steps of validation and confirmation of primary hits, we employed a secondary screen with DRC and TAL analysis.

Plate reformatting of 87 primary hits was done manually and 5 concentrations of fivefold dilutions (from 5 μΜ to 8 nM) of each small molecule was tested in n=4 replicates as before. Live neuronal numbers were fitted to dose-response curves versus the logarithmic scale of chemical molecules concentrations (x axes) and plotted into groups based on their chemical fingerprint clustering. Results showed a wide variety of DRC (**Fig.S3**) which were classified according to previous studies [74, 75]. We did not obtain any false positive hits from our primary screening assay, since all 87 primary hits were still active at 5 μM. The n=4 replication, the proper z-score cut off and the z-scores standard deviation consideration in selection criteria of primary hits, could be the explanation. We obtained a group of highly active molecules (4 Dose Active) that reversed the MCOS induced toxicity at four highest concentrations. Among them was Resveratrol with well documented neuroprotective activities and numerous targets in the antioxidant pathway [132, 133].

However, almost half of primary hits were excluded from further validation since they were active only at the highest concentration, or had partial curves and poor efficacies. For example Minocycline, a second-generation tetracycline (TC) which showed high efficacy only at 5 μM was excluded as a single dose active molecule. The initial library included other TCs drugs which showed some potency in reversing the neurotoxicity but all were below the cut off used in the primary screen assay. It is known that TC have direct scavenging activity towards free radicals [134] while Minocycline particularly, was found to be neuroprotective in experimental ischemic stroke model and Iron Induced Brain Injury models [135].

In a retrospective Total Axonal Length analysis of DRC experiment we further eliminated several chemicals from the primary hits list. One of them was the β-blocker

Carvedilol, which was named as the most potent free radical scavenger among other βblockers [136]. The IC50 of Carvedilol in inhibiting (Fe^2+^)-initiated lipid peroxidation in rat brain homogenate was determined at 8.1 μM. [136], 3.1 μM higher than the concentration we used. This could be the explanation why Carvedilol was not efficient enough in reversing the MCOS induced ferroptosis by retaining intact axonal network in TAL analysis and could not reach out the highest asymptote at 5 μM concentration in DRC experiment.


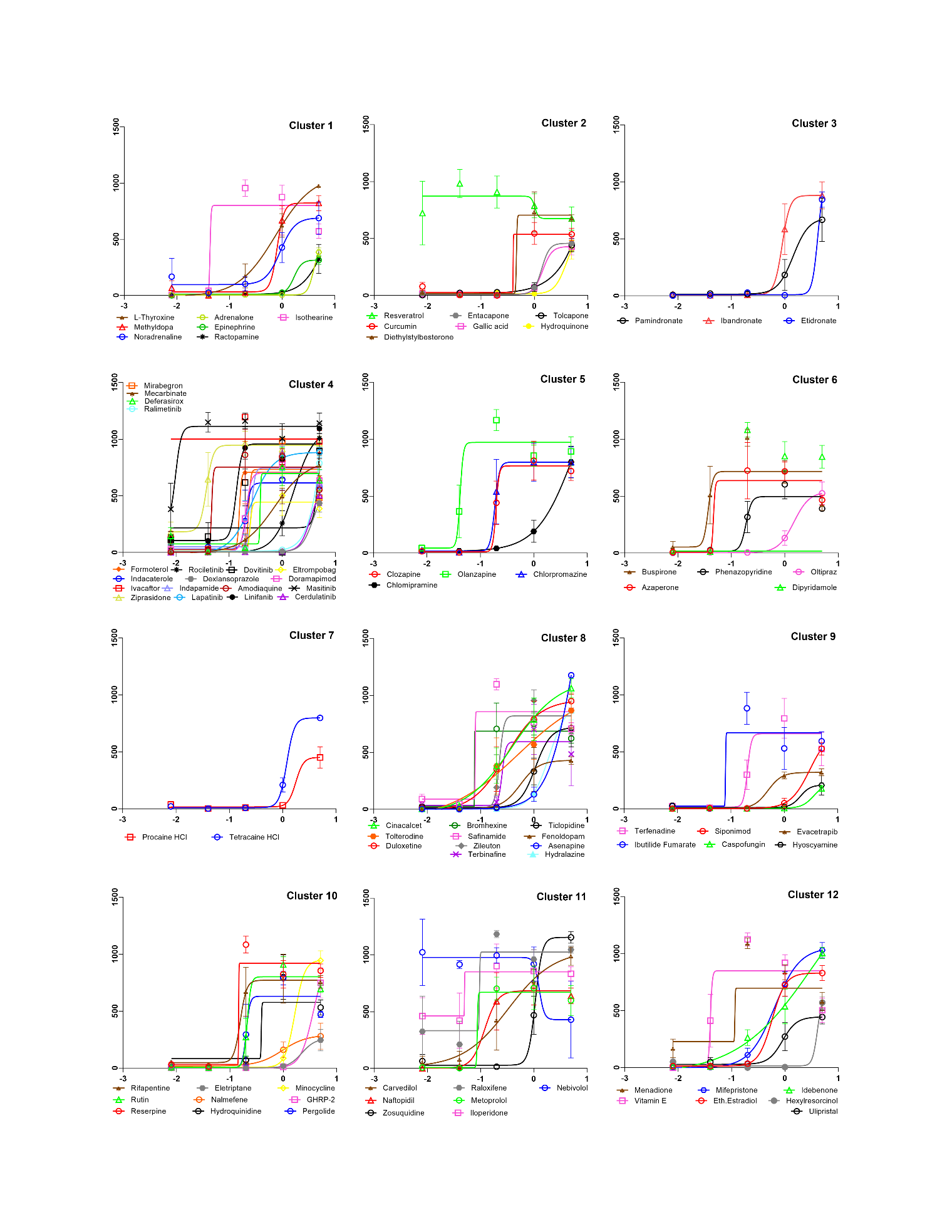


**Fig.S3: Dose Response Curves of primary hits**: Half-log concentration intervals from 5μM to 8nM in n=4 replicate plates were tested. Drugs were added as before on day6 -5wAO treated (H1 diku) neurons. Results were plotted (GraphPad Prism v9.3.1) per chemical structure clusters 1 to 12. Log scale of concentration on x axes and live neuronal counts on y axes as determined by CellProfiler Analyst (v4.0.0 Aug 2021). Data are the mean and ±SEM.

**Reference for Material and methods continue from main manuscript.**

1. Zhang C, Deng Y, Dai H, Zhou W, Tian J, Bing G, et al. Effects of dimethyl sulfoxide on the morphology and viability of primary cultured neurons and astrocytes. Brain Res Bull. 2017 Jan;128:34-39. doi: 10.1016/j.brainresbull.2016.11.004. Epub 2016 Nov 9. PMID: 27836802.
2. Zhang JH, Chung TD, Oldenburg KR. A Simple Statistical Parameter for Use in Evaluation and Validation of High Throughput Screening Assays. J Biomol Screen. 1999;4(2):67-73. doi: 10.1177/108705719900400206. PMID: 10838414.
3. Lee KH, Cha M, Lee BH. Neuroprotective Effect of Antioxidants in the Brain. Int J Mol Sci. 2020 Sep 28;21(19):7152. doi: 10.3390/ijms21197152. PMID: 32998277; PMCID: PMC7582347.
4. Allgrove J. Biphosphonates. Arch Dis Child. 1997 Jan;76(1):73-5. doi: 10.1136/adc.76.1.73. PMID: 9059169; PMCID: PMC1717046.
5. Dombrecht EJ, Cos P, Vanden Berghe D, Van Offel JF, Schuerwegh AJ, Bridts CH, Stevens WJ, et al. Selective in vitro antioxidant properties of bisphosphonates. Biochem Biophys Res Commun. 2004 Feb 13;314(3):675-80. doi: 10.1016/j.bbrc.2003.12.149. PMID: 14741688.
6. Singh N, Agrawal M, Doré S. Neuroprotective properties and mechanisms of resveratrol in in vitro and in vivo experimental cerebral stroke models. ACS Chem Neurosci. 2013 Aug 21;4(8):1151-62. doi: 10.1021/cn400094w. Epub 2013 Jun 24. PMID: 23758534; PMCID: PMC3750679.
7. Gu T, Wang N, Wu T, Ge Q, Chen L, Antioxidative Stress Mechanisms behind Resveratrol: A Multidimensional Analysis, Review Article Hindawi Journal of Food Quality, 2021, Article ID 5571733,<https://doi.org/10.1155/2021/5571733>
8. Kładna A, Michalska T, Berczyński P, Kruk I, Aboul-Enein HY. Evaluation of the antioxidant activity of tetracycline antibiotics in vitro. Luminescence. 2012 JulAug;27(4):249-55. doi: 10.1002/bio.1339. Epub 2011 Aug 23. PMID: 22887986.
9. Guo J, Chen Q, Tang J, Zhang J, Tao Y, Li L, et al. Minocycline-induced attenuation of iron overload and brain injury after experimental germinal matrix hemorrhage. Brain Res. 2015 Jan 12;1594:115-24. doi: 10.1016/j.brainres.2014.10.046. Epub 2014 Oct 31. PMID: 25451129.
10. Yue TL, Cheng HY, Lysko PG, McKenna PJ, Feuerstein R, Gu JL, et al. Carvedilol, a new vasodilator and beta adrenoceptor antagonist, is an antioxidant and free radical scavenger. J Pharmacol Exp Ther. 1992 Oct;263(1):928. PMID: 1357162.

**Supplementary Figure Legends**

**Fig.S1: Differential susceptibility of H1-H2 haplotypes to MCOS induced axonal degeneration and neuronal death.** (**A**) Cytotoxicity assay with three cell lines (names in the upper panel) per haplotype, the H1, H2 and the heterozygous H1/H2. NPCs were treated and differentiated to neurons with (solid bars) and without AO (stripped bars) for indicated weeks. Relative fluorescence units of Calcein-AM on day10 (green bars) and days12/13 (gray bars) of neuronal maturation. Unpaired t-test comparison of plus to minus AO treated cell lines from two independent experimental replicates. Values are mean and ±SEM. P-values are 0.1234 (ns), 0.0332(*), 0.0021(**), 0.0002(***). (**B-C**) Representative fields of bright field images from H1 cell line (diku) NPCs treated with plusAO (B) and -5wAO (C). (**D** to **G**) Differences of axonal degeneration and neuronal death in representative fields of bright field images from day8 neurons of H1 (diku, D,E) and H2 (uilk, F,G) with plusAO (left column) and -5wAO (right column). Scale bar 100 pixels on ImageJ. (**H** to **M**) Representative fields of neuronal death spreading in population. Time lapse for 24h (1fph) of tiled images from nine fields and DEAD\LIVE kit (red/green). Upper row H1 (diku, yemz, lepk), lower row H2 haplotype (qolg, zihe, uilk) at -5wAO depletion. H-I imaged on day9, J-K-L day12 and M day21 of neuronal differentiation. Scale bar 100μm. For complete time-lapse movies refer to supplementary videos vS09 to vS14 respectively.

**Fig.S2: FDA-approved chemical library screening with MCOS sensitive H1 neurons.** (**A**) Representative field of day12 neurons imaged with Calcein-AM (Red) of negative (no rescue on day6) and positive (day6 rescue) controls. (**B**) Live neuronal counts on days 8, 10 ,12 and 14 of neuronal maturation from -5wAO treated H1 neurons (diku) and rescued with AO supplementation on day3 and day6. Rescue with full AO and diluted to 1/25 and 1/625 on day6 were tested in three experimental replicates. Results are the mean and ± SEM compared with one-way ANOVA and Sidak's post Hoc test. (**C**) The maximum DMSO concentration which is tolerable by H1 neurons under MCOS on day6 rescue was determined to be the 0.5%. since the 1% of DMSO is significantly different from no DMSO added on day6 rescue. Results are the mean and ± SEM compared with one-way ANOVA and Sidak's post Hoc test. P-values are 0.1234 (ns), 0.0332(*), 0.0021(**), 0.0002(***). (**D**) Primary screening assay quality measurement with plate wise z-factors from n=4 replicates per screening plate (HST1 to HTS29) based on positive and negative controls. (**E**) n=4 replicates correlation plot including a linear regression (black line) with corresponding R2 correlation factor from primary screen analysis with HitSeekR.

**Table S1: Chemical structure classification of primary hits.** Clusters 1 to 7 after Exact SAHN clustering of primary hits based on their DayLight fingerprints and Ward's linkage method (Scaffold Hunter v2.6.3). Chemical structures of primary hits based on Chemical Entities of Biological Interest (ChEBI) ontology (May, 2022) are shown. Molecular targets and drug indications given by Selleckhem with classifications from ChEBI ontology (Jan, 2022).

**Table S2: Chemical structure classification of primary hits.** Clusters 8 to 12 of primary hits based on their DayLight fingerprints (Scaffold Hunter v2.6.3). Chemical structures from Chemical Entities of Biological Interest (ChEBI) ontology (May, 2022), molecular targets and drug indications given by Selleckhem and classifications from ChEBI ontology (Jan, 2022).

**Fig.S3: Dose Response Curves of primary hits**: Half-log concentration intervals from 5μM to 8nM in n=4 replicate plates were tested. Drugs were added as before on day6 -5wAO treated (H1 diku) neurons. Results were plotted (GraphPad Prism v9.3.1) per chemical structure clusters 1 to 12. Log scale of concentration on x axes and live neuronal counts on y axes as determined by CellProfiler Analyst (v4.0.0 Aug 2021). Data are the mean and ±SEM.

**Supplementary videos**

vS01_D09F14dikuday8+5wAOTL5h: H1 diku treated with plusAO corresp. **Fig.1E**

vS02_C04F04dikuday8-5wAOTL5h: H1 diku treated -5wAO corresp. **Fig.1F**

vS03_F09F07uilkday8+ 5wAOTL5h: H2 uilk treated with plusAO corresp. **Fig.1G**

vS04_G03F02uilkday8- 5wAOTL5h: H2 uilk treated -5wAO corresp. **Fig.1H**

vS05_3dplotPC1-2-3d8CP90 Ellips_movie corresp. **Fig.4D**

vS06_3dplotPC1-4-5d8CP90 Ellips_movie corresp. **Fig.4E**

vS07_3dplotPC1-2-3-d9CP90 Ellips_movie no corresp. Fig

vS08_3dplotPC1-4-5d9CP90 Ellips_movie no corresp. Fig

vS09_20250203_135252B08F04-5wAOd09diku: H1 corresp. **Fig.S1H** -5wAO day9

vS10_20250203_143408C07F05-5wAOd08yemz: H1 corresp. **Fig.S1I** -5wAO day8

vS11_20250203_141958D10F07-5wAOd09lepk: H1 corresp. **Fig.S1J** -5wAO day9

vS12_20250203_151104E11F06-5wAOd12qolg: H2 corresp. **Fig.S1K** -5wAO day12

vS13_20250203_151428F07F05-5wAOd12zihe: H2 corresp. **Fig.S1L** -5wAO day12

vS14_20250203_153135G11F07-5wAOd21uilk: H2 corresp. **Fig.S1M** -5wAO day21
